# Chikungunya Fever: A Clinical and Virological Investigation of Outpatients on Reunion Island, South-West Indian Ocean

**DOI:** 10.1371/journal.pntd.0002004

**Published:** 2013-01-17

**Authors:** Simon-Djamel Thiberville, Veronique Boisson, Jean Gaudart, Fabrice Simon, Antoine Flahault, Xavier de Lamballerie

**Affiliations:** 1 UMR 190, Emergence des Pathologies Virales, Aix-Marseille Univ-IRD-EHESP French School of Public Health, University Hospital Institute for Infectious Disease and Tropical Medicine, Marseille, France; 2 Infectious Disease and Tropical Medicine, AP-HM CHU Nord, University Hospital Institute for Infectious Disease and Tropical Medicine, Marseille, France; 3 Department of Medical Intensive Care, CHU South Site, Saint Pierre, Reunion, France; 4 Aix-Marseille Univ, UMR192, SESSTIM (AMU, IRD, INSERM), Marseille, France; 5 Department of Infectious Diseases and Tropical Medicine, Laveran Military Teaching Hospital, Marseille, France; 6 EHESP French School of Public Health, Rennes-Sorbonne Paris Cité, Paris, France; University of Texas Medical Branch, United States of America

## Abstract

**Background:**

Chikungunya virus (CHIKV) is responsible for acute febrile polyarthralgia and, in a proportion of cases, severe complications including chronic arthritis. CHIKV has spread recently in East Africa, South-West Indian Ocean, South-Asia and autochthonous cases have been reported in Europe. Although almost all patients are outpatients, medical investigations mainly focused on hospitalised patients.

**Methodology/Principal Findings:**

Here, we detail clinico-biological characteristics of Chikungunya (CHIK) outpatients in Reunion Island (2006). 76 outpatients with febrile arthralgia diagnosed within less than 48 hours were included by general practitioners during the CuraChik clinical trial. CHIK was confirmed in 54 patients and excluded in 22. A detailed clinical and biological follow-up was organised, that included analysis of viral intrahost diversity and telephone survey until day 300. The evolution of acute CHIK included 2 stages: the ‘viral stage’ (day 1–day 4) was associated with rapid decrease of viraemia and improvement of clinical presentation; the ‘convalescent stage’ (day 5–day 14) was associated with no detectable viraemia but a slower clinical improvement. Women and elderly had a significantly higher number of arthralgia at inclusion and at day 300. Based on the study clinico-biological dataset, scores for CHIK diagnosis in patients with recent febrile acute polyarthralgia were elaborated using arthralgia on hands and wrists, a minor or absent myalgia and the presence of lymphopenia (<1G/L) as major orientation criteria. Finally, we observed that CHIKV intra-host genetic diversity increased over time and that a higher viral amino-acid complexity at the acute stage was associated with increased number of arthralgia and intensity of sequelae at day 300.

**Conclusions/Significance:**

This study provided a detailed picture of clinico-biological CHIK evolution at the acute phase of the disease, allowed the elaboration of scores to assist CHIK diagnosis and investigated for the first time the impact of viral intra-host genetic diversity on the disease course.

## Author Summary

The mosquito-transmitted chikungunya virus is responsible for acute febrile polyarthralgia and, in a proportion of cases, complications including chronic arthritis. Since 2005, it has massively re-emerged in the Old World. Although the large majority of patients are outpatients, the most detailed studies have focused previously on hospitalised patients (*i.e.*, severe cases). Here, we report the detailed clinico-biological characteristics of ‘standard’ clinical presentations in patients followed-up by general practitioners in Reunion Island (2006) during the CuraChik clinical trial. At the onset of the disease, two stages were observed: *(i)* a ‘viral stage’ during the first 4 days, associated with an acute febrile polyarthralgic syndrome and a subsequent rapid clinical improvement; the main clinico-biological characteristics during that period were used to elaborate supportive chikungunya diagnostic scores, (*ii*) a ‘convalescent stage’ (days 5–14) with no detectable viraemia but a slower clinical improvement. Woman and elderly patients were found at risk for more symptomatic forms of the disease at both the acute and late stages (day 300) and we observed that the viral intra-host genetic diversity increased over time and that a higher viral amino-acid complexity at the acute stage was associated with more symptomatic illness at the late stage of the disease.

## Introduction

Chikungunya virus (CHIKV) is an arbovirus (genus *Alphavirus*, family *Togaviridae*), transmitted by the bite of infected mosquitoes (*Aedes aegypti* = *Stegomya aegypti* and *Aedes albopictus* = *Stegomya albopicta*), that causes Chikungunya fever (CHIK), an acute febrile illness characterized by severe and often debilitating arthralgia [1]. CHIKV was first isolated in 1952 in East-Africa [2]. The name ‘Chikungunya’ refers to the stooped posture that develops in an individual as a result of arthritic symptoms and comes from the Bantu language of the Makonde people (Tanzania and Mozambique) [2]. Until recently, knowledge of CHIK clinical features was based on its first description in the 1970s [3], [4]. Since 2005, CHIK outbreaks of unprecedented magnitude have occurred in South-Asia and the Indian Ocean islands, including Reunion Island with an estimated 266,000 cases, accounting for roughly one third of the population [5]. In 2007, a viraemic traveller from India introduced CHIKV into northern Italy, resulting in an outbreak with 292 suspected cases [6]. Thus, CHIK constitutes a serious threat numerous tropical and temperate areas due to the fact that *Aedes albopictus* is now widespread, notably in Southern-Europe [6], [7], and displays seasonal synchronicity within potential endemic areas [8].

Although almost all CHIK patients are outpatients, most clinical and laboratory investigations of CHIK focused on hospitalised patients (*i.e.* mostly with severe presentations that represented a very small proportion of total infections) [9], [10], [11], [12]. Our attempts to describe the clinical and biological features of chikungunya acute disease took advantage of data collected during the CuraChik clinical trial, performed on Reunion Island during the 2006 Indian Ocean outbreak [13]. CuraChik provided a unique opportunity to collect detailed clinical and biological information from CHIKV infected patients with the most common forms of clinical presentation, recruited by general practitioners.

## Materials and Methods

### Objectives

- We aimed to provide a precise clinical and biological description of acute laboratory-confirmed CHIKV infection in outpatients and some information regarding follow-up until day 300.
- We also compared CHIKV positive and negative patients recruited on the basis of clinical presentation with acute febrile arthralgia during an epidemic period.
- We finally performed a comprehensive comparative analysis of intra-host viral genetic diversity.

### Study design

#### Patients and procedures

The main details of the CuraChik trial have been reported elsewhere [14]. Curachik (http://clinicaltrials.gov/ct2/show/NCT00391313) was a randomised double blind, placebo-controlled, prospective trial aiming at evaluating the efficacy and safety of chloroquine as therapeutic treatment of CHIK **(study protocol available on File S1)**.

This trial included adult patients (18–65 years old, men and women), who volunteered to take part in the study, residing in Reunion Island, having a typical presentation of acute CHIK (defined by acute febrile arthralgia) diagnosed within less than 48 hours. Exclusively general practitioners (GPs) enrolled the eligible patients.

Clinical data were collected from three sources: *(i)* a daily auto-questionnaire from day 1 (D1) to day 14 (D14); *(ii)* three consultations with a GP on D1, day 7 (D7) (mean 6.4, SD = 1.4) and day 25 (D25) (mean 26.5, SD = 9.8); *(iii)* a telephone questionnaire on day 100 (D100) (mean 130.5, SD = 28.1) and day 300 (D300) (mean 300.7, SD = 13.8).

Biological data were collected from the analysis of blood samples on D1, D3, D6 and D16. The extraction of nucleic acids and CHIKV specific RT-PCR [15] were carried out from all samples (D1, D3, D6 and D16). Tests for the presence of CHIKV-specific immunoglobulin G (IgG) and IgM were performed at D1, D6 and D16 by an indirect immunofluorescence assay (according to a procedure previously described [16]).

The case definition of CHIKV positive patients relied on the association of a CHIKV specific positive RT-PCR on D1 and seroconversion on D16. CHIK negative patients tested negative for CHIKV genome on D1 and showed no evidence of seroconversion on D16.

#### Ethics Statement

The study commenced on May 20, 2006, after obtaining authorisation from the French Health Products Safety Agency and Ethics Committee. All subjects provided informed written consent.

#### Intra-host viral genetic diversity

Ten patients with a variety of clinical and biological presentations and a positive CHIKV RT-PCR diagnostic test were selected within the placebo subgroup. The extraction of nucleic acids was performed from D1 and D3 sera using the EZ1 virus MiniKit (virus card 2.0) and an EZ1 biorobot (Qiagen, Germany) according to the manufacturer's protocol. For all patients (n = 10), a 692-nucleotide fragment within the E1 gene (positions 10138–10829) was amplified using the high fidelity Eppendorf One-Step RT-PCR kit and primers CV1F (5′-CTATCGCTTGATTACATCACG-3′) and CV1R (5′-CGCTTCCGGTATGTCGATG-3′). This fragment includes amino acidsspanning position E1/226. The mutation A226V has been reported to confer a virus adaptation to *Aedes albopictus* [17], [18]. Analysis was also performed for samples collected at D3 for 2 patients still viraemic at this time. Amplification products were purified (QIAquick PCR Purification Kit), subjected to 3′ end adenosine addition (*Taq* polymerase, Invitrogen), ligated into the cloning vector pCR 2.1 and transformed into TOP10 competent cells, according to the manufacturer's protocol (TA Cloning, Invitrogen). An average of 45 clones per serum sample was sequenced directly from the plasmid using the T7 promoter primer (5′-CCCTATAGTGAGTCGTATTA-3′).

Sequences were analysed with the Sequencher software and aligned with ClustalX [19]. For a given sample, the sequence of each clone was compared with the most common sequence using the Mega 4.1 programme [20]. Differences in nucleotide and protein sequences were analysed according to different clinical, biological or demographic parameters. Sequence divergence was evaluated using the pairwise distance among nucleotide (π nt) and amino acid (π aa) sequences. The mean ratio of non-synonymous (dN) to synonymous (dS) substitutions per site was estimated using the pairwise method of Nei and Gojobori [21] in MEGA. For the analysis of intra-host genetic diversity, the sequence of each clone was compared to all other clones for each human serum. The percentage of variable nucleotide sites (number of variable nt sites/number of nt sites), of nucleotide mutations (number of nt mutation/number of nt sequenced), and of mutant clones (number of clones with mutation/total number of clones) was calculated, as well as the π nt, π aa, dN, dS and dN/dS parameters.

#### Statistical analysis and diagnostic score

To assess factors relating to clinical presentation and laboratory abnormality during CHIK on D1 and D300, we performed univariate analysis, for qualitative factors with Fisher's exact test and for continuous factors using the Mann Whitney nonparametric test. Correlations were assessed using the Spearman nonparametric test. Following the Mickey and Greenland approach [22], variables with P-values <0,2 were retained and entered into backwards stepwise multivariate linear regression models (logistic, Gaussian or Poisson regression depending on the dependent variable, respective percentage, continuous or count variable), following the Mickey and Greenland approach [22]. The standard deviation (SD) and 95% confidence interval (CI) of the odds ratio (OR) (logistic regression), the linear regression coefficient (β) (Gaussian regression) and the Standardized Incidence Ratio (SIR, Poisson regression) were estimated.

To compare the intra-host diversity and the available clinical and laboratory data, we performed correlation analyses using the Spearman nonparametric test.

Finally, we evaluated the possibility of generating a diagnostic score of CHIK on the first day of monitoring, by comparing the clinical and biological features of CHIKV positive and negative samples. Any factor for which a Pearson's chi2 or Fisher test was <0.2, was included in multivariate analysis (hierarchical log-linear model) to study the adjusted relationship between different variables and their interactions. Sensitivities (Se), specificities (Sp), predictive values - positive (PPV) and negative (NPV) - were estimated and yielded a Receiver Operating Characteristic (ROC) curve and the area under the curve (AUC).

All statistical analyses were performed with the IBM SPSS statistic 19 software.

For multivariate analysis, the alpha probability threshold of significance was 0.05.

## Results

### Population studied

Amongst 76 patients included at D1, the diagnosis of CHIKV was confirmed in 54 patients (CHIKV+ve patients) and excluded in 22 patients (CHIKV−ve patients). Since the clinical and biological assessment at D1 was obtained prior to the beginning of the treatment, all CHIKV+ve patients could be used for analysis at the time of inclusion. By contrast, only the patients who received the placebo (27 CHIKV+ve and 13 patients CHIKV−ve, placebo group) were used to describe the evolution of the disease.

#### Clinical presentation at inclusion

Amongst CHIKV+ve patients (54 patients), the mean age was 40 years old, the sex ratio (m/f) was 1.7 and the mean weight was 76.1 kg **(** **Table 1** **)**. The average time between symptom onset and inclusion was 1.2 days (ranging between 0–2, SD = 0.595) reflecting the initial stage of illness. Five (9.3%) patients had pre-existing orthopaedic illness.

**Table 1:** Clinical and biological characteristics of Curachik patients*.

	CHIKV+ n (%)	CHIKV− n (%)	p-Value	Odd Ratio (95% CI)
**Age**				
Mean (SD, min-max)	40.1 (12.4, 18–66)	41.4 (15.3, 20–66)	0.92	
18–20	4 (7.4)	1 (4.5)		
21–30	9 (16.7)	9 (40.9)		
31–40	19 (35.2)	2 (9.1)		
41–50	10 (18.5)	3 (13.6)		
51–60	8 (14.8)	3 (13.6)		
61–66	4 (7.4)	4 (18.2)		
**Gender N (%)**				
Male	34 (63)	11 (50)	0.3	
Female	20 (37)	11 (50)		
**pre-existing orthopaedic illness**	5 (9.3)	5 (22.7)	0.1	
**Localisation of Arthralgia**				
Metacarpophalangean (MCP)	40 (74.1)	9 (40.9)	**<0.01**	0.24 (0.09;0.69)
Interphalangean (PIP)	37 (68.5)	9 (40.9)	**0.038**	0.32 (0.11;0.89)
Hands (MCP+PIP)	43 (79.6)	10 (45.5)	**<0.01**	0.21 (0.07;0.62)
Wrist (W)	39 (72.2)	8 (36.4)	**<0.01**	0.22 (0.07;0.63)
Ankles	37 (68.5)	11 (50)	0.2	
Knees	33 (61.1)	17 (77.3)	0.2	
Shoulders	26 (48.1)	11 (50)	1.	
Lombalgia	25 (46.3)	12 (54.5)	0.6	
Feet	23 (42.6)	4 (18.2)	**0.06**	0.30 (0.089;1.01)
Cervicalgia	21 (38.9)	9 (40.9)	1.	
Elbows	14 (25.9)	10 (45.5)	0.11	
**Myalgia**				
moderate or important	25 (46.3)	18 (81.8)	**<0.01**	5.22 (1.56;17.48)
**Neutropenia (<2 G/l)**	12 (22.2)	2 (10)	0.32	
**Lymphocytes (<1G/l)**	43 (79.6)	4 (20)	**<0.001**	0.06 (0.02;0.23)
Mean (SD, min-max)	0.8 (0.57, 0.3–4)	1.5 (0,59, 0.6–2.7)	**<0.001**	
**Platelets (<150G/l)**	14 (25.9)	1 (5)	**0.055**	0.15 (0.018;1.23)
Mean (SD, min-max)	185 (58.5, 104–348)	217 (54,5, 148–362)	**0.014**	
**C-Reactive Protein (CRP) (mg/l)**				
Mean (SD, min-max)	52.4 (42.4, 7–195)	51.8 (63.8, 0–264)	0.26	
**ASAT/ALAT >45 UI/l**	11 (22)	0 (0)	**0.028**	0.78 (0.67;0.9)

* *This trial included adult patients (18–65 years old) with a typical presentation of acute chikungunya disease (defined by acute febrile arthralgia) diagnosed within less than 48 hours. Exclusively general practitioners (GPs) enrolled the eligible patients, during the 2006 CHIKV outbreak on Reunion Island.*

*The case definition of CHIKV positive patients relied on the association of a chikungunya-specific positive RT-PCR on D1 and seroconversion on D16 ; the case definition of CHIKV negative patients relied on the association of a chikungunya-specific negative RT-PCR on D1 and negative serology on D16. Laboratory confirmed chikungunya cases (CHIKV+, 54 patients) were compared with those excluded (CHIKV−, 22 patients).*

The most common presenting clinical symptom was a febrile poly-arthralgia (16 joints on average, out of 34 on the proposed diagram, SD = 10, median = 17) with an intensity assessed as important (among 4 categories: absent, minimal, moderate, important) in 46.3%. Arthralgia was symmetrical (*i.e.*, bilateral) in 53.7% of cases and small joints were more frequently affected (hands 79.6%, wrist 72.2%, ankles 68.5%, knees 61.1%, lumbar 46.3%, shoulders 48.1%, feet 42.6%, cervix 38.9%, elbows 25.9%) **(** **Table 1** **)**. No arthralgia lateralization according to right-handed or left-handed was noted. General symptoms were most often recorded as headache (72%), asthenia (76%), myalgia (74%) and chills (83%).

**Figure 1:**
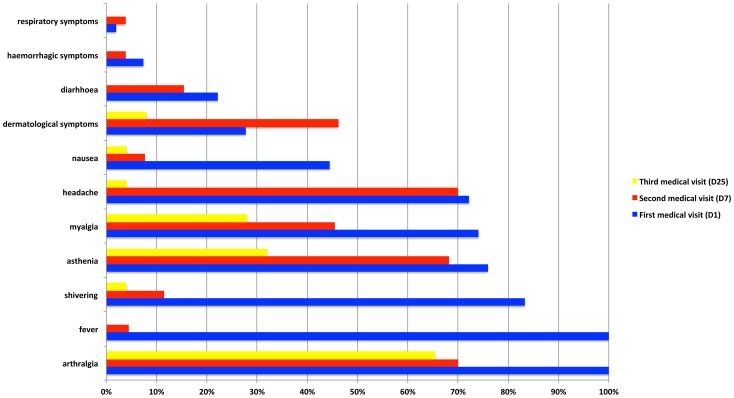
Clinical features of ambulatory CHIKV+ve patients* at days 1, 7 and 25. These clinical data were collected from three consultations with a general practitioner on day 1, day 7 (mean 6.4, SD = 1.4) and day 25 (mean 26.5, SD = 9.8) of the disease during the Reunion island outbreak 2005–2006. * Since clinical assessment during the first medical visit was obtained prior to the beginning of the treatment, all CHIKV+ve patients (N = 54) could be used for analysis. By contrast, only patients who received the placebo (N = 27) were included in the study of disease evolution (second and third medical visits).

Few other signs were noted: dermatological signs (28%), digestive disorders such as nausea/vomiting (44%), diarrhoea (22%) and dysgeusia (13%) **(** **Figure 1** **)**.

**Figure 2:**
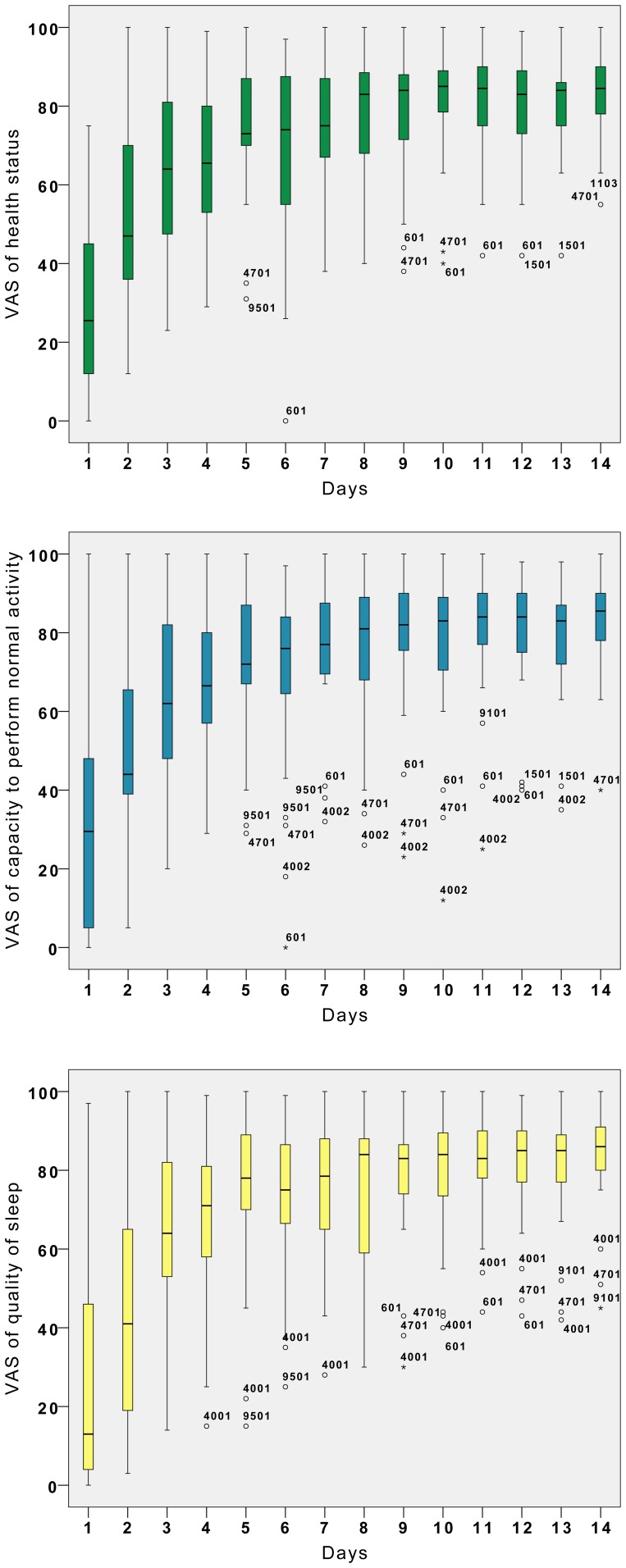
Quality of life assessed by ambulatory CHIKV+ve patients* from day 1 until day 14. Three kind of quality of life (health status, capacity to perform normal activity and quality of sleep) were assessed by self reported visual analogic scale (VAS) from “very bad” (VAS = 0) to “very good” (VAS = 100) and are represented here by box plot diagrams. Box plot is a representative diagram of continuous variables. The bottom and the top of the box are the 25^th^ and 75^th^ percentile, the band near the middle is the median and the ends of the whiskers are the 1.5 inter-quartile of the lower and upper quartile. The data not included between the whiskers are plotted as an outlier with small circles (if between 1.5 to 3 inter-quartile of the lower or upper quartile) or with a star (if higher than 3 inter-quartile of the lower or upper quartile). The outliers are tagged with their patient numbers to follow them at different time period. * Clinical assessment at D1 was obtained from all CHIKV+ve patients (N = 54) during the Reunion island outbreak 2005–2006. Only patients receiving placebo (N = 27) were included in D2–D14 clinical assessment.

Quality of life (assessed by visual analogical scales (VAS)) was severely impacted with the capacity to perform normal activities, health status and quality of sleep assessed on average at 33/100 (SD = 26), 28/100 (SD = 25) and 27/100 (SD = 13), respectively. More than half of the patients scored <30/100 in all aspects of quality of life evaluation **(** **Figure 2** **)**.

Demographic and biological data were compared as functions of clinical presentation at D1 using multivariate analysis. A higher number of arthralgic joints was independently associated with women (p<0.001, SIR = 1.361, CI (1.191;1.554)), with an increase of age (p<0.001, SIR = 1.017, CI (1.011;1.022)) and a decrease of leukocytosis (p<0.001, SIR = 0.881 CI(0.841;0.923)). Patients with important arthralgia were significantly older than others (p<0.05, OR = 1.073, CI (1.013;1.136)) and associated with a shorter time between onset of symptom and inclusion (p<0.05, OR = 0.288, CI (0.071;0.764)). Regarding quality of life no significant association, in multivariate analysis with any risk factor analysed, could be identified.

### Biological presentation at inclusion

Lymphopenia was frequent at inclusion (94% of cases with a value <1.5 Giga per Litre (G/L); 79.6% with a value <1 G/L). Thrombocytopenia (<150 G/L) was noted in 24% of cases and neutropenia (<2.5 G/L, but always >1 G/L) in 33% of cases. Abnormal liver function (ALT >45 International Unit per Litre (IU/L) and AST >35 IU/L) was found in 14% and 28% respectively. C Reactive Protein (CRP) was >15 mg/L in 82% of cases, >50 mg/L in 33% and >100 mg/L in 12% **(** **Table 1** **)**. There was no leukocytosis (>10 G/L). Five patients had anaemia (<12 g/dl), including one patient had sickle disease (8 g/dl).

The average viral load at D1 was 1.2×10^9^ (3.7×10^5^–1.4×10^10^, SD = 2.3×10^9^ RNA copies/ml).

In multivariate analysis, a lower lymphocytosis was associated with a shorter time between onset of symptoms and inclusion (p = 0.053, β = 0.249, CI(−0.03;0.502)) and a higher viral load (p<0.05, β = −0.144, CI(−0.284;−0.004)). A higher viral load was associated with an increase of age (p<0.05, β = 0.024, CI(0.001;0.047)) and a decrease of delay of inclusion (p<0.05, β = −0.608, CI(−1.093;−0.124)).

#### Clinical and biological evolution

There was no hospitalisation during the 14-day follow-up and two clinical stages of evolution were observed **(** **Figures 2** **and** **3** **)**:

**Figure 3:**
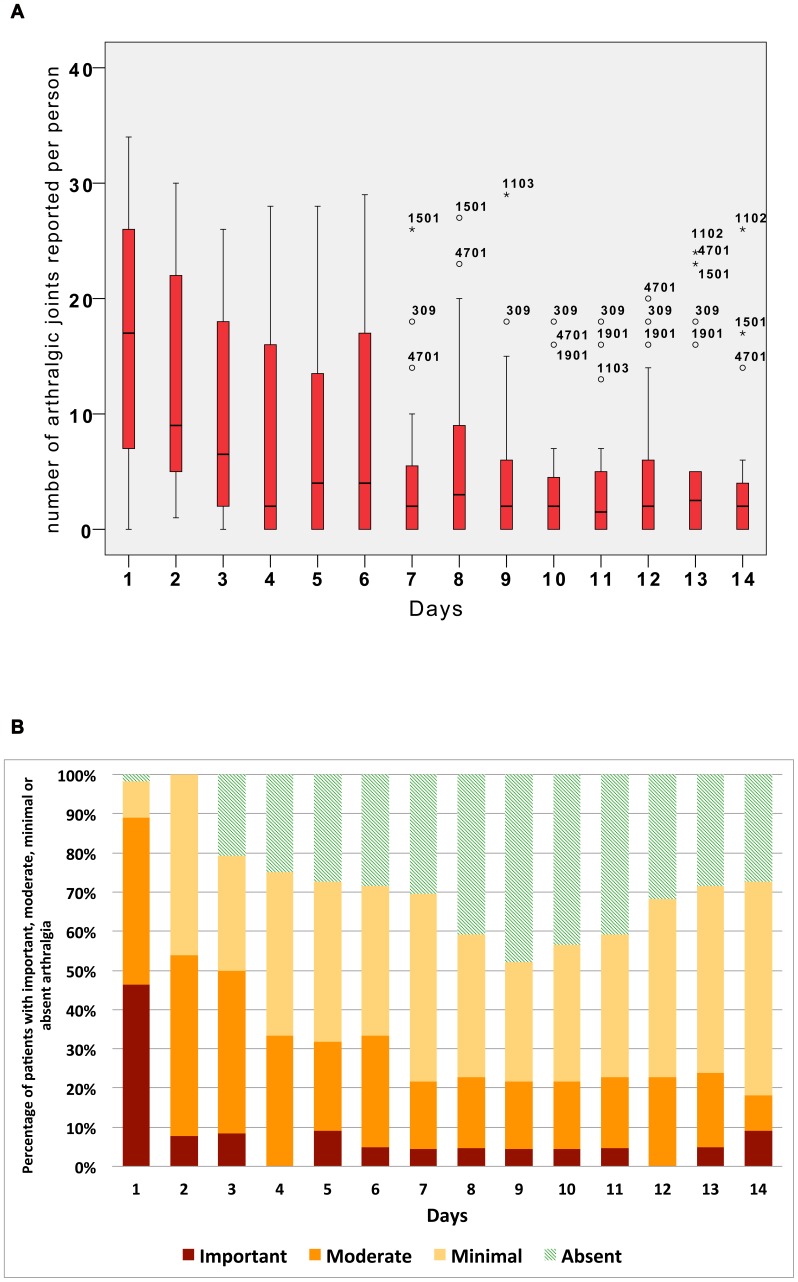
Number and intensity of arthralgic joints for CHIKV+ve* patients. **Figure 3A**: Self reported number of arthralgic joints reported per person (day 1–day 14). **Figure 3B**: Percentage of patients with absent, minimal, moderate or severe arthralgia (day 1–day 14). * Characteristics of patients included and details of box plots are the same as reported in *figure 2* *.*

- From D1 to D4, a rapid improvement was observed (the percentage of patients with arthralgia decreased from 98% to 68%, the average number of arthralgic joints decreased from 16 to 9 (SD = 8,7), VAS quality of life scores increased more than twice and the percentage of VAS <50/100 decreased from 83% to 19%).
- From D5 until D14, the evolution was slower (68% of patient still suffered from arthralgia on D14 with an average number of 6 painful joints per person (SD = 7,2), VAS quality of life was assessed at 82/100 on average, with less than 5% of VAS <50).

At the last medical visit (D25), 36% of patients reported residual asthenia; 65% reported arthralgia (corresponding to 71% with sporadic and 57% with permanent arthralgia respectively) while 44% and 32% of patients were assessed by GPs as recovered or improved in condition respectively.

The number of patients with positive viraemia decreased from 54 (100%) on D1 to 21 (39.6%) on D3 and the average viral load was 6.42 (4.43 to 9, SD = 1.19, log10 copies/ml). In all cases there was a decrease of viraemia from D1 to D3. At D6, all viral loads were negative. At D16, there was neither lymphopenia nor thrombocytopenia, while neutropenia and abnormal liver function persisted in 12% and 7% of patients respectively. CRP was >5 mg/L in 12% of cases without exceeding 50 mg/L.

During the D300 telephone interview, 5 out of 26 patients who could be contacted (19.2%) considered that they had not completely recovered; 6/26 (23%) declared residual arthralgia (7 joints on average, SD = 5.3); morning stiffness was assessed in 4/6 (66.7%) cases but arthralgia was always related to joint activity. Retrospectively, the duration of initial illness was estimated at more than 4 weeks by 12 out 21 patients who considered that they had completely recovered (57.1%). In univariate analysis, patients who did not report recovery at D300 were older (p<0.05, 52.80±12.61 *vs* 35.62±10.56) and more frequently women (p<0.05, women 60.0% (3/5); men 9.5% (2/21)). Patients who reported persistent arthralgia at D300 were older (p<0.01, 51.33±11.84 *vs* 35.20±10.65) and had a higher number of arthralgic joints at D1 (p<0.05, 23.50±6.75 *vs* 12.25±9.86). In multivariate analysis, the number of painful joints at D300 was higher for women (p = 0.065, SIR = 1.841, CI (0.963;3.520)) and increased with age (p<0.001, SIR = 1.142, CI (1.103;1.183)).

#### Diagnostic score, comparison between CHIKV+ve and CHIKV−ve patients

Analysis of the CHIKV+ve and CHIKV−ve groups at baseline revealed no difference in the distribution of age, sex, weight or time of inclusion **(** **Table 1** **)**. In univariate analysis, CHIKV+ve patients had:

- more frequent pain in small upper joints (wrists, W) (p<0.01, OR = 4.55, CI(1.59;13.04)), metacarpophalangeal (MCP) (p<0.01, OR = 4.13, CI(1.45;11.74), proximal interphalangeal (PIP) (p<0.05, OR = 3.14 CI(1.13;8.77)),
- more frequently minor or absent myalgia (MYOPAIN) (p<0.01, OR = 5.22, CI(1.56;17.48))
- more frequently lymphopenia (<1 G/L) (p<0.0001, OR = 15.64, CI(4.35;56.25)).

Factors that showed a direct relationship in univariate and multivariate analyzes with CHIK diagnosis were W, MCP, MYOPAIN and lymphopenia. CHIKV+ve and CHIKV−ve patients were not statistically different for the impact on quality of life at the acute stage.

Finally, the probability (p) of having been infected by CHIKV has been estimated, using logistic regression, as follows:

p = 1/(1+exp(4.139)×exp(−3.666×lymphopenia)×exp(−1.940×MCP+)×exp(−2.341×W+)×exp(−2.700×MYOPAIN)). Each covariate was validated if equal to 1, otherwise 0. Tested on our cohort, the ROC curve had an AUC = 0.93 (p<0.001), with Se = 90%, Sp = 85%, PPV = 94% and NPV = 77% using a 0.579 probability threshold value.

Similarly, we could calculate a clinical score relying only on clinical symptoms (*i.e.*, excluding lymphopenia) : p = 1/(1+exp(1.609)×exp(−1.450×MCP+)×exp(−1.732×W+)×exp(−2.044×MYOPAIN)). Tested on our cohort, the ROC curve had an AUC = 0.81 (p<0.001) and with Se = 76%, Sp = 73%, PPV = 87% and NPV = 55% using a 0.717 probability threshold value.

Noticeably, in both scores, the variable Handpain (pain on MCP and/or PIP) could be used instead of the MCP variable with similar results.

**Figure 4:**
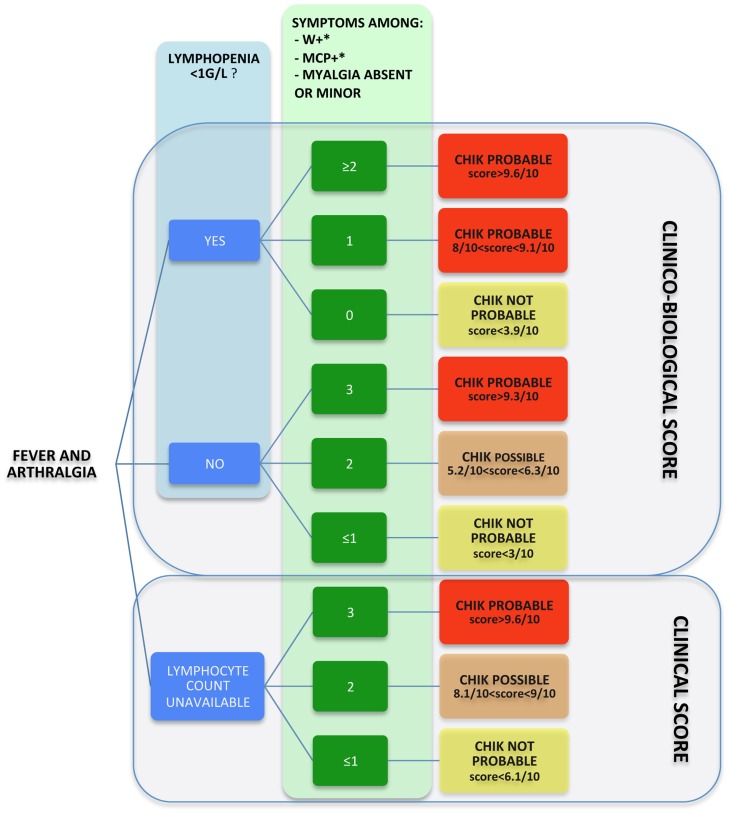
Clinical and clinico-biological score for the diagnosis of CHIK. These scores were based on patients who reported fever and arthralgia for less than 48 hours. * MCP+: arthralgia on at least one metacarpophalangeal joint. * W+: arthralgia on at least one wrist. We propose a clinical score based exclusively on clinical data (W+, MCP+ and Myalgia absent or minor) and a clinico-biological score that further includes lymphopenia (<1G/L). The result of the score represents the predicted probability to have chikungunya (see calculation of scores in the main text).

Finally, these results were used to propose a clinical and clinico-biological score usable in ambulatory practice by GPs (see on **Figure 4**). The values of the score presented in Figure 4 represented, in our cohort, the predicted probability of having been infected by CHIKV, with each score.

#### Intra-host viral genetic diversity

To investigate the intra-host genetic diversity of CHIKV we sequenced, on average, 45 clones/serum (41–57) within the E1 gene **(** **Table 2** **)**. For the 10 D1 sera, the average proportion of mutant clones observed was 35.6%, the mutations occurred in 10 (1.4%) to 27 (3.9%) sites of the 692 nucleotides sequenced and the average proportion of non-synonymous mutations was 73.2%. Forty-eight (24%) mutations were observed more than once (41 twice, 7 in three clones). The transition/transversion ratio was 5.8 (219∶38). The average dN/dS ratio (that illustrates selective pressure [23]), was 1.03 and 0.16 at the intra-host and inter-host level, respectively. Over a total of 519 clones studied, the A226V mutation was found in all but one clone (99.8%) and in-frame stop codons were identified in 2 clones (0.4%).

**Table 2:** Intra-host genetic diversity analysis of CHIKV populations deciphered from 10 sera of CHIKV+ve patients.

CHIKV samples #ID nb, D1 or D3, sex/age, time to inclusion, viral load, number of arthralgia at day 300	N° of clonestested	% of mutant clones	% of variable nt sites	% of nt mutations	π nt	π aa	d_N_	d_S_	d_N_/d_S_
**#304, D1, M/56 years, 1 day, 9.3 log10/ml, 0**	47	27.66%	2.02%	0.05%	0.0009	0.0022	0.00098	0.0005	1.93
**#304, D3, M/56 years, 1 day, 9.3 log10/ml**	40	32.5%	1.45%	0.05%	0.0010	0.0024	0.00104	0.0006	1.74
**#4002, D1, F/28 years, 0 day, 9.3 log10/ml, 0**	40	24.4%	1.73%	0.04%	0.0008	0.0019	0.00084	0.0009	0.96
**#4002, D3, F/28 years, 0 day, 9.3 log10/ml**	38	42.5%	3.18%	0.09%	0.0018	0.0041	0.00180	0.0018	1.00
**#302, D1, F/26 years, 1 day, 9.0 log10/ml, 0**	43	37.21%	2.89%	0.07%	0.0013	0.0026	0.00116	0.002	0.59
**#1103, D1, F/40 years, 2 days, 7.3 log10/ml (NA)**	45	39.13%	2.89%	0.06%	0.0013	0.0030	0.00133	0.0011	1.28
**#1201, D1, M/44 years, 2 days, 6.7 log10/ml, 4**	40	45.10%	3.9%	0.08%	0.0016	0.0029	0.00129	0.0024	0.55
**#1501, D1, F/62 years, 2 days, 7.0 log10/ml, 12**	43	41.30%	3.61%	0.09%	0.0018	0.0039	0.00174	0.0021	0.84
**#3001, D1, M/52 years, 2 days, 8.1 log10/ml, 0**	43	37.21%	2.46%	0.08%	0.0015	0.0036	0.00159	0.0014	1.15
**#4001, D1, M/40 years, 1 day, 8.4 log10/ml, 0**	53	29.82%	3.32%	0.06%	0.0012	0.0028	0.00125	0.0013	1.00
**#4102, D1, M/30 years, 1 day, 5.6 log10/ml, 0**	45	40.43%	3.61%	0.08%	0.0017	0.0026	0.00115	0.0031	0.37
**#4701, D1, M/66 years, 2 days, 7.0 log10/ml, 12**	42	33.33%	2.75%	0.07%	0.0013	0.0031	0.00137	0.0008	1.59

The percentage of variable nucleotide (nt) sites was calculated as the number of variable nt sites ×100 divided by the number of nt analysed (758 nt).

The percentage of nucleotide (nt) mutations was calculated as the number of nt mutations ×100 divided by the number of nt sequenced for each serum sample.

The average pairwise distance was calculated among the nucleotide (p nt) and amino acid (p aa) sequences from each serum.

The mean ratios of non-synonymous (dN) and synonymous (dS) substitutions per site were estimated using the pairwise method of Nei and Gojobori.

Sex: Male: male; F: female.

NA: not available.

Analysis of CHIKV intra-host genetic diversity was conducted according to demographic, clinical and biological data **(** **Table 2** **)**. The major findings were:

- An increased percentage of mutant clones and average π aa and dN was significantly correlated with an increased delay between onset of disease and inclusion (p = 0.045 for % of mutant clones, p<0.001 for π aa and dN).
- A high viral load at D1 was significantly correlated with a low intra-host diversity (p<0.01 for % of mutant clones, % of nt mutations, % of variable nt sites and π nt, p<0.05 for dS).
- A comparison of intra-host genetic diversity based on sequential serum at D1 and D3 from the same patients (n° 304 and n°4002) revealed an increase of genetic diversity over time **(** **Table 2** **)**.
- From the D300 telephone interview, intensity of sequelae and an increased number of reported arthralgia were significantly correlated with a higher amino-acid diversity at inclusion (π aa, dN; p<0.05).

## Discussion

Here we have reported a prospective study on Reunion Island of 54 adult outpatients, examined by general practitioners during the 2006 CHIK outbreak. These outpatients represent ‘standard’, ‘mild’, clinical presentations (the basis for inclusion was a recent presentation associating fever and arthralgia), which did not require hospitalisation or specific treatment for complications. Such patients represent the majority of cases: the proportion of hospitalised patients during the CHIKV 2005–2006 outbreak in Reunion Island was estimated to be 0.3% [12] whilst the majority of medical investigations were dedicated to hospitalised patients. Thus our knowledge of severe presentations is currently more accurate than that of common presentations that do not require hospitalisation.

On Reunion Island, the epidemiological surveillance system was based, at first, on active and retrospective case detection around the reported cases, and then relied on a sentinel network of general practitioners [24]. Accordingly, some clinical data regarding outpatients could be collected from cases notified through either the epidemiological surveillance system [24], [25], [26], or community based cross sectional survey [27], [28], [29], [30], [31], [32], [33]. However, the unique aspect of the current study relies on the detailed clinical and virological follow-up of such patients. It included laboratory confirmation of CHIKV infection (based on RT-PCR and seroconversion), a clinical follow-up at the acute phase (associating daily auto-questionnaires and medical consultations), and late assessments (by telephone until day 300). The study took place at the end of the outbreak (*i.e.*, the enrolment was performed by practitioners who had previously managed approximately one third of the population infected by the virus), and there is no evidence that another arbovirus (*e.g.* dengue virus) has been circulating in Reunion Island during this period.

This study has obvious limitations. Firstly, a low patient count: 54 patients, with a confirmed CHIKV infection, was studied on day 1 (inclusion), but the follow-up was performed for only 27 of these patients, corresponding to the placebo arm of the clinical trial. Secondly we could not exclude a potential impact of a placebo effect during the follow-up. Thirdly, 22 patients with a negative diagnosis of CHIK (of whom 13 received placebo) were also included but, regarding outpatients and mild presentations, the aetiology of their disease could not be further investigated. Finally, the number of patients for which in depth analysis of intra-host viral genetic diversity was analysed was low (10 patients) and, despite interesting and statistically significant results, this specific aspect will deserve in the future analysis from a larger cohort.

### Assessment at inclusion

The clinical presentation of CHIK at inclusion revealed a quite severe impact of the disease on quality of life, with more than half of the patients' scores <30/100. It conformed with the canonical presentation previously reported in Reunion Island and in the recent Indian reports, which included fever and symmetrical poly-arthralgia [9], [10], [34], [35], [36], [37], [38]. However, this simplistic association (fever+polyarthralgia) seems to perform modestly for the specific diagnosis of CHIK: in a retrospective serologic survey of the CHIK outbreak in Mayotte Island [28], the PPV was as low as 74%. In our study, despite the great recent clinical experience of general practitioners, the PPV was similar (71%).

Looking into further details, it appears that arthralgia was most commonly observed in small joints (*i.e.*, wrists, ankles, hands) and knees, as reported from both in- and outpatients [9], [10], [29], [34], [39]. More precisely, this study highlighted the massive number of arthralgic joints (16, on average) and the specific importance of *(i)* small joint pain such as MCP/PIP or wrist and *(ii)* a minor or absent myalgia for the diagnosis of CHIK at the onset of the disease.

This strongly suggests that a convenient diagnostic score may profitably guide the diagnosis of CHIK at the acute stage of the disease. We proposed a very simple and purely clinical score (**Figure 4**) which reached 87% PPV in our population (*i.e.*, outpatient 18 to 60 years old, examined before the second day of illness). For convenience, results were categorised as ‘probable’, ‘possible’ and ‘not probable’.

Our data also highlighted the high level of viral load (1.2×10^9^ RNA copies/ml on average) at inclusion. It was slightly higher than in other reports [10], [40], possibly due in the current study, to the very short delay between onset of disease and inclusion. Viral load at D1 significantly increased with age but no relationship with clinical presentation or co-morbidity could be identified. In contrast with previous studies dedicated to hospitalised patients [9], [41], we did not identify a relationship between the level of CRP and transaminases, a reduction of the polymorphonuclear neutrophil level or other biological abnormalities, and the intensity and number of arthralgia or the quality of life at D1. However, as previously reported from inpatients [10], we observed that lymphopenia (<1G/L) was closely related to the level of viraemia. It constituted an important clue for the diagnosis of CHIK as illustrated by our clinico-biological score (**Figure 4**), which again, classified the patients as ‘probable’, ‘possible’ or ‘not probable’, as a function of the probability to be infected by CHIKV, based on clinical presentation and lymphocyte count. The PPV of this score reached 94% in our population, for a threshold of 0.579.

### Evolution of the disease

The most original input from the CuraChik protocol was the detailed information collected (patient self-assessment from D1 to D14, medical consultations (D1, D7, D25), biological analyses (D1, D6, D16)), which altogether provided an accurate description of the evolution of patients during the acute stage of the disease.

Deciphering these data indicated that the acute disease includes 2 distinct stages

- the first (D1–D4, ‘viral stage’) was associated with viraemia, *i.e.*, clinical symptoms that reflect the viral ‘burst’ and the initiation of innate immunity, associated with high levels of pro-inflammatory cytokines [42], [43], [44] such as interferon-α and IL-6 but also IL-1Ra, IL-12, IL-15, IP-10 and MCP-1. This is in coherence with results from Chow et al., who extended the ‘acute phase’ of the disease until D4 [44]. In all cases in the current study, this response was associated with a rapid decrease of viremia from D1 to D3 (39.6% of patients remained viraemic at D3). In parallel, the clinical presentation improved promptly: the daily self-assessment, showed a rapid decrease of the number of arthralgic joints (from 16 to 9) **(** **Figure 3a** **)** and all quality of life parameters dramatically improved **(** **Figure 2** **)**.
- From day 5 to day 14 (‘convalescent stage’), all patients had no detectable viraemia, but improvement was slower, considering both quality of life scores **(** **Figure 2** **)** or rheumatic parameters **(** **Figure 3a, 3b** **and supporting information figure S1 and movie S1)**. The initial symptoms (*e.g.*, fever and shiver that fell from >80% at D1 to 10% at D7) receded but by the D7 medical visit >40% of patients had persistent arthralgia, asthenia, myalgia or headache. Dermatological signs were more often identified on D7 than D1 **(** **Figure 1** **)**, in coherence with studies reporting skin lesions mostly after disease onset [45], [46], [47]. This observation may provide an explanation for the large range of frequency of dermatological signs in the literature (from 10% to 86%) [4], [30]. Previous reports [44] showed that, by day 10, a number of inflammatory mediators (including interferon-α, IL-6, IL-1Ra, IL-12, IL-15, IP-10 and MCP-1) had significantly decreased, at least in patients with initial high viraemia.

However, despite clinical improvement, it is probable that immune mechanisms are still involved [48] at this stage. The final outcome (complete clinical recovery or persistent pain and chronic joint inflammation) appears likely to depend upon a series of genetic, viral and immunologic factors that operate at the acute and convalescent stages. On Reunion Island [31], [43] and India [39] but not Singapore [44], late complications were associated with severe acute disease. Here, in agreement with the clinical pattern observed on Reunion Island, patients with a high number of arthralgic joints at disease onset reported more frequently persistent arthralgia at D300.

The early and convalescent immune response may be, in addition to putative yet uncharacterised viral factors, modulated by innate (genetic) and acquired factors. The latter certainly include age, which appears in many studies to be a major determinant of the clinical presentation and outcome. Here, we found that an increase of age was an independent risk factor for symptomatic illness at the time of disease onset (number and intensivity of joint pains) and at D300 (number of cases with persistent arthralgia). At D300, the patients who did not report recovery and who reported persistent arthralgia were significantly older. These results are consistent with studies on hospitalised patients and Indian report which reported that elderly patients more frequently presented with atypical feature or a severe course [10], [49], [50] with persistent arthralgia [31], [39], [43], [49], [50], [51].

Genetic factors presumably trigger different immune responses which may account for the inter-individual and inter-ethnic variability of clinical presentation. Amongst them, gender is of specific interest. A single report mentioned a higher susceptibility of males, to CHIKV infection [52] but globally, previously published data suggest that symptomatic CHIK is more frequent in women [35], [38]. On Reunion Island, women were over-represented based on reported cases [24], [25] whereas cross sectional studies, based on representative groups of the population, found similar seroprevalence values in females and males [32], [53]. In two other studies, at the late stage of the disease, female gender was associated with persistent arthralgia [41] or light cerebral disorder or fatigue [27]. In the current study, females were independently associated with a high number of painful joints at disease onset and at D300. They also reported non-recovery more frequently at D300. It is difficult at this stage to distinguish between gender-related specific clinical susceptibility and a different perception of the disease in males and females.

The interplay between the immune response and viral evolution most probably constitutes an important issue for disease outcome. A non-primate animal model [42] showed that CHIKV could persist much longer than previously believed and such persistence may imply the existence of specific adapted variants, or at least depend on the kinetics of viral clearance by the immune system. To our knowledge, no study had previously described the intra-host genetic diversity of CHIKV in human samples. We observed that CHIKV was represented in serum by a variety of closely related genomes and that genetic diversity increased over time and was correlated with the decrease of viral load. This observation relied on the analysis of sequential serum samples from 2 patients, but was also supported by the analysis of other sera for which analysis of the intra-host genetic diversity of CHIKV was made available: the later the serum was sampled, the higher was the intra-host genetic diversity, based on percentage of mutant clones and average π aa and dN. These data are consistent with a mechanism in which acute infection produced an accumulation of mutations over time (resulting in an increased intra-host genetic diversity), associated with a lower number of virions and, possibly, an increased potential for persistence. Interestingly, we found that a higher amino-acid complexity at the acute stage was associated with increased reporting of arthralgia and intensity of sequelae at D300. This may indicate that the immunological processes associated with the initial viraemia decline or are partly circumvented, thus enhancing the opportunity for onset of virus persistence and long term clinical complications. In an experimental model, Coffey *et al.* previously observed that a CHIKV variant with high fidelity polymerase produced truncated viraemia and lower organ titres and suggested that reduced genetic diversity impacts negatively on virus fitness in both invertebrate and vertebrate hosts [54]. Moreover in the macaque model, long-term CHIKV infection was observed in joints, muscles, lymphoid organs, and liver which could explain the long-lasting CHIK symptoms observed in humans [42]. Altogether, these data are consistent with the hypothesis that CHIKV displays increased intra-host diversity which may be associated with prolonged viraemia, higher organ viral load and an increased risk of chronic disease. If this is the case, such mechanisms appear to be quite different from those previously observed in the case of dengue fever, where lower intra-host diversity has been associated with more severe cases [55] On the other hand, our results appear more closely related to previous reports on the relationship between intra-host genetic diversity, fitness and virulence in the examples of chronic infections by induced HIV or HCV [56], [57]. Some additional studies are needed to further characterise the intra-host genetic diversity of CHIKV at the acute phase of the disease. Sequential analysis provided by New Generation Sequencing tools may provide a more accurate picture of this diversity and allow a powerful analysis of the relationship between the structure and evolution of intra-host viral genetic diversity and the clinical evolution of CHIKV infected patients.

## Supporting Information

File S1 **CuraChik protocol.** CuraChik was a randomised double blind, placebo-controlled, prospective trial aiming at evaluating the efficacy and safety of chloroquine as therapeutic treatment of CHIK (http://clinicaltrials.gov/ct2/show/NCT00391313).
(PDF)

Figure S1 **Evolution of the percentage of arthralgia and health status during the acute stage of CHIK.** The size of the disc for a given joint represents the percentage of arthralgic patient for this specific joint. The colours of the human figure represent the mean of the health status of the population studied. The right column represents the morning assessment from day 2 until day 5 and the left column represents the evening assessment from day 2 until day 14. Clinical assessment at D1 was obtained from all CHIKV+ve patients (N = 54). Only patients receiving placebo (N = 27) were included in D2–D14 clinical assessment.
(TIF)

Movie S1 **Evolution of the percentage of arthralgia and health status during the acute stage of CHIK.** The size of the disc for a given joint represents the percentage of arthralgic patient for this specific joint. The colours of the human figure represent the mean of the health status of the population studied. Only the evening assessments from day 1 to day 14 were included within this movie. Clinical assessment at D1 was obtained from all CHIKV+ve patients (N = 54). Only patients receiving placebo (N = 27) were included in D2–D14 clinical assessment.
(MOV)
