# Supplementary material for: Chikungunya Fever: A Clinical and Virological Investigation of Outpatients on Reunion Island, South-West Indian Ocean
Source: PLoS Negl Trop Dis. 2013 Jan 17;7(1):e2004. doi: 10.1371/journal.pntd.0002004 (PMC3547841; doi:10.1371/journal.pntd.0002004)
Supplement: File S1 — CuraChik protocol. CuraChik was a randomised double blind, placebo-controlled, prospective trial aiming at evaluating the efficacy and safety of chloroquine as therapeutic treatment of CHIK (http://clinicaltrials.gov/ct2/show/NCT00391313). (PDF) [file pntd.0002004.s001.pdf]

PROTOCOLE D'ESSAI RANDOMISÉ

CURACHIK

Essai randomisé en double insu comparant la chloroquine au placebo dans le traitement curatif de la maladie du chikungunya

CODE DE L'ETUDE : CURACHIK

Comité scientifique de l'essai

Promoteur :
Assistance Publique Hôpitaux de Marseille (APHM)

Investigateur principal :
Xavier de Lamballerie (APHM, université de la Méditerranée)

Co-investigateurs principaux :
Bernard Alex Gaüzère (St Denis, CHD, île de la Réunion)
Véronique Boisson (St Pierre, GHSR, île de la Réunion)
Sophie Journeaux (Ste Marie, Médecine Générale, île de la Réunion)
Philippe De Chazournes (Président de Medocéan, île de la Réunion)

Méthodologie
APHM
Antoine Flahault (Inserm-UPMC UMR-S707)

Contrôle qualité et audit
APHM
François Favier (CIC-La Réunion)

Pharmaciens référents
CHD, Service de la Pharmacie (Françoise Chan Ou Teung)
GHSR, Service de la Pharmacie (Nicolas Istria)

Comité indépendant de suivi de l'essai
Epidémiologie : Mark Wilson, Ann Arbor, USA
Infectiologie : José Miro, Barcelona, Spain
Virologie : Alan Hay, Mill Hill, London, UK

Les produits délivrés dans cet essai sont fournis par les laboratoires Sanofi-Aventis

Sommaire

1 RESUME SYNOPTIQUE DU PROTOCOLE 3
2 SCHEMA DE L'ETUDE 5
3 INTRODUCTION ET JUSTIFICATION DE L'ETUDE 7
4 OBJECTIFS DE L'ETUDE 8
4.1 Objectifs principal 8
4.2 Objectifs secondaires 8
5 PLAN EXPERIMENTAL 8
5.1 Type de l'étude 8
5.2 Durée de l'étude 9
5.3 Nombre prévu de sujets 9
5.4 Critères d'éligibilité pour une éventuelle inclusion 9
5.5 Critères d'inclusion 9
5.6 Critères de non-inclusion 9
5.7 Evaluation pré-thérapeutique 10
5.8 Visite d'entrée 10
5.8.1 Evaluation clinique 10
5.8.2 Evaluation biologique 10
5.9 Randomisation et désignation du numéro des sujets 10
5.10 Traitements étudiés 10
5.11 Administration des médicaments étudiés 10
5.12 Suivi des patients et recueil des données 11
5.12.1 Suivi des patients 11
5.12.2 Recueil des données 11
5.12.3 Réseaux de médecins libéraux 11
5.12.4 Information des sujets 11
5.12.5 Liste des données recueillies 11
5.12.6 Randomisation et mise en œuvre des traitements 12
6 STATISTIQUES 12
6.1 Variables d'analyse 12
6.2 Méthodes d'analyse statistique 13
6.2.1 Comparabilité des groupes 13
6.2.2 Analyse des critères principaux 13
6.2.3 Analyse des critères secondaires 14
6.2.4 Analyse médico-économique 14
6.3 Justification du nombre de sujets 14
7 JUSTIFICATION DU RECOURS A DES DONNEES INDIRECTEMENT NOMINATIVES 14
8 JUSTIFICATION DE L'APPLICATION DE LA LOI HURIET 15
9 SURVEILLANCE DES EVENEMENTS INDESIRABLES 15
9.1 Surveillance des événements indésirables et des événements indésirables graves 15
9.2 Définition des événements indésirables (EI) et des événements indésirables graves (EIG) 15
9.3 Obligations de l'investigateur en matière de déclaration des événements indésirables 16
9.4 Grossesse 16
9.5 Obligations du promoteur 17
10 CONSIDERATIONS ETHIQUES ET LEGALES 17
10.1 Cadre réglementaire de l'étude 17
10.2 Obligations légales 17
10.3 Information des sujets 17
10.4 Protection des données personnelles 17
10.5 Comité indépendant de suivi de l'essai 17
10.6 Arrêt prématuré de l'étude 17
11 MONITORAGE ET CONTROLE DES DONNEES 19
12 DOCUMENTATION ET UTILISATION DES RESULTATS DE L'ETUDE 19
12.1 Documentation des résultats de l'étude 19
12.2 Utilisation des résultats de l'étude 19
13 DUREE DE L'ETUDE ET CALENDRIER 20
14 ANNEXES 20
14.1 Engagements du promoteur et de l'investigateur 20
14.2 Cahier d'observation 20
14.2.1 Informations recueillies par le médecin généraliste investigateur 20
14.2.2 Données du bilan standard et du test de grossesse 20
14.2.3 Informations recueillies par autoquestionnaire (patient) 20
14.2.4 Informations recueillies par l'équipe mobile de monitorage 20

1 RESUME SYNOPTIQUE DU PROTOCOLE

CuraChik : Essai randomisé en double insu comparant la chloroquine au placebo dans le traitement curatif du chikungunya

Contexte
Une sévère épidémie de fièvre de Chikungunya a été observée sur l'île de la Réunion depuis le début de l'année 2006. L'agent étiologique est le virus Chikungunya, un arbovirus du genre *Alphavirus*, transmis par la piqûre de moustiques (sur l'île de la Réunion il s'agit du moustique *Aedes albopictus*). A ce jour, plus de 210,000 cas ont été recensés. La plupart correspondent à des pathologies bénignes, avec fièvre et arthralgies intenses, mais des complications majeures (encéphalites, hépatites..) ayant nécessité une hospitalisation dans un service de réanimation ont été rapportées pour 85 cas de diagnostic confirmé. De plus, 34 cas d'infections néonatales ont été documentés. Enfin, 148 certificats de décès mentionnent le terme "Chikungunya" comme cause probable de la mort.
Il n'existe aujourd'hui aucun traitement antiviral contre le virus Chikungunya. Nous avons montré par des études *ex-vivo* (dans un modèle de cultures cellulaires susceptibles à l'infection virale) que la chloroquine avait un pouvoir inhibiteur significatif vis à vis de la réplication du virus Chikungunya. Cette efficacité est retrouvée à des doses correspondant aux doses plasmatiques observées chez des patients traités contre le paludisme avec cette molécule.

Nous proposons de tester l'efficacité de la chloroquine dans le traitement de l'infection humaine par le virus Chikungunya. Il s'agit d'un essai thérapeutique avec bénéfice individuel direct.

Objectifs
Comparer expérimentalement en double insu à la Réunion en termes d’efficacité et de tolérance la chloroquine au placebo dans le traitement curatif du chikungunya.

Population concernée
Patients adultes de plus de 18 ans et moins de 66 ans (hommes et femmes non enceintes, sans contre-indications) volontaires pour participer à l’essai comportant 5 jours de traitement curatif, résidant à la Réunion, ayant un poids corporel supérieur ou égal à 60 kilogrammes et chez lequel un médecin généraliste investigateur de l’essai a porté un diagnostic de chikungunya clinique évoluant depuis moins de 48 heures.

Recrutement
Il sera réalisé en premier lieu par les médecins du réseau Medocéan (environ 160 généralistes répartis sur l'île de la Réunion). Les médecins des urgences du CHD et du GHSR, ainsi que les médecins généralistes qui seraient volontaires pour rejoindre l'essai pourront également inclure des patients, en coordination avec le réseau Medocéan.

Méthodes
Plan expérimental
Essai randomisé en double insu testant l’efficacité entre deux groupes de 125 patients. Les patients atteints de chikungunya clinique sélectionnés par les médecins généralistes de l’étude seront stratifiés selon le binôme (= infirmière + TEC), et la région. L’essai comparera en deux bras l’efficacité et la tolérance de la chloroquine contre placebo dans le traitement curatif du chikungunya clinique aigu.
- **Bras 1** : chloroquine *per os*
    - 600 mg le premier jour en une prise [soit 6 gélules de 100 mg] (J1)
    - deux prises quotidiennes de 300 mg [soit 3 gélules de 100 mg] pendant 2 jours (J2 &J3)
    - une prise quotidienne de 300 mg [soit 3 gélules de 100 mg] le soir pendant 2 jours (J4 & J5)
- **Bras 2** : placebo *per os*
    - 600 mg le premier jour en une prise [soit 6 gélules de 100 mg] (J1)
    - deux prises quotidiennes de 300 mg [soit 3 gélules de 100 mg] pendant 2 jours (J2 &J3)
    - une prise quotidienne de 300 mg [soit 3 gélules de 100 mg] le soir pendant 2 jours (J4 & J5)

Recueil de données
- Recrutement des patients par le médecin généraliste et recueil des éléments cliniques pertinents le jour du diagnostic (J1), à l'exception des critères principaux de jugement et de ceux donnant lieu à quantification par auto-questionnaire remis au patient. Consultation du médecin généraliste à J7 (recueil de données à l'exception des critères principaux de jugement et de ceux donnant lieu à quantification par auto-questionnaire remis au patient) et J25 (recueil de données complet, fin du protocole). Pourront rester suivis (surveillance observationnelle par le médecin traitant dans le cadre d'une enquête) les patients qui ne seront pas totalement rétablis à J25.

- Recueil portant sur les signes cliniques (par auto-questionnaire) auprès des patients des deux bras, à J1 en présence du TEC, puis deux fois par jour jusqu'à J5 et une fois par jour de J6 à J14.

Critères de jugement :
Le critère principal de l'essai en analyse en intention de traiter est l'efficacité en termes de réduction de la durée des symptômes cardinaux (fièvre et arthralgies).

Les critères secondaires sont :
- En analyse "per protocole", la réduction de l'impact sur la virémie (durée et intensité).
- la durée et la sévérité de chacun des symptômes non cardinaux, les événements indésirables notifiés dans les deux bras de l'essai, l'observance thérapeutique, le nombre et la durée des arrêts de travail et la consommation d'antalgiques et d'anti-inflammatoires.
- Sur le plan économique l'essai permettra d'évaluer le rapport coûts/bénéfices de la chloroquine par rapport à l'absence de traitement ; l'évolution de la charge virale et la chloroquinémie seront étudiées chez tous les patients jusqu'à J16.

Méthodes statistiques
L'analyse principale sera réalisée en intention de traiter sur l'ensemble de la population des patients ayant un Chikungunya suspecté cliniquement, et ayant au moins reçu une dose de chloroquine (ou de placebo). Après une description des deux groupes sur les variables avant randomisation, et vérification de l'équilibre entre les groupes, l'analyse portera sur le critère principal, par un test de Mantel-Haenszel stratifié sur le binôme (= infirmière + TEC).
L'analyse des critères secondaires sera réalisée avec le test exact de Fisher pour les variables qualitatives et de Wilcoxon modifié pour tenir compte de la stratification par binôme, pour les variables continues (en particulier la valeur de la virémie à l'inclusion).
Autres analyses : une analyse sur la population des patients randomisés ayant un chikungunya clinique virologiquement confirmé (par culture cellulaire, RT-PCR ou séroconversion) et ayant au moins reçu une dose sera réalisée. Deux analyses "per protocole" porteront sur les deux populations définies ci-dessus.

Nombre de sujets nécessaires
Un effectif de 100 sujets par bras de randomisation, soit au total 200 sujets remplissant les critères d'inclusion sera nécessaire pour mettre en évidence une différence entre le bras expérimental (chloroquine) par rapport au bras de référence (placebo) de deux jours sur la durée des symptômes (pour un écart-type de 5 jours) avec une puissance de 80 %, pour un test en formulation bilatérale avec un seuil de signification $P < 0,05$.
Le risque de diagnostic clinique erroné (faux positifs) pouvant dans les premières études réalisées à la Réunion avoisiner 20%, l'effectif final a été fixé à 125 sujets par bras, soit 250 au total.

L'observation d'une réduction de 2 jours de la durée des symptômes se justifie par la brièveté habituelle de la pathologie dans sa forme non-compliquée (une semaine environ).

Le tirage au sort sera réalisé par Sanofi-Aventis. Cette recherche sera soumise aux avis favorables préalables du CPP, de la CNIL et de l'AFSSAPS.

Déroulement
Inclusion des patients de plus de 18 ans et moins de 66 ans volontaires consultant pour une maladie de chikungunya évoluant depuis moins de 48 heures. Patients ayant présenté les signes de chikungunya dans le passé exclus.
Contre-indications à la chloroquine exclus (voir détails au paragraphe 5.6). Femme enceinte ou allaitante exclue Prélèvements à domicile pour documentation de l'infection chez l'ensemble des patients (bilan standard, RT-PCR, isolement du virus sur culture cellulaire, chloroquinémie, étude de la réponse immune dont sérologie).
Randomisation, attribution des traitements.
Durée de traitement : 5 jours à domicile.
Recueil des données: Prélèvements à J1, J3, J6 et J16 (infirmière à domicile). Recueil des données d'efficacité et de tolérance par autoquestionnaire et consultation à J7 et J25. Les prélèvements seront transmis au laboratoire compétent du GHSR et du CHD pour le bilan standard et les analyses spécialisées. Centralisation des sérums au laboratoire de référence de l'essai (hôpital de la Timone, Marseille) qui transmettra un aliquot aux CNR des arbovirus et des fièvres hémorragiques (Lyon). Test des chloroquinémies, isolement des souches en culture cellulaire et étude de la réponse immune (hors sérologies) par le laboratoire de référence de l'essai (hôpital de la Timone, Marseille).

Durée de l'étude et calendrier
La durée de l'étude pour un patient est de 25 jours, le jour de l'inclusion (J1) correspondant au premier jour du traitement.
Les inclusions dans l'essai sont prévues après l'obtention des accords administratifs et financiers, au mieux durant la saison 2006, avant l'hiver austral. Il sera proposé aux médecins généralistes investigateurs de la Réunion (réseau MEDOCEAN) de randomiser les 250 patients. Un régulateur en charge de l'essai communiquera tous les jours de la période d'inclusion l'état des inclusions ; un médecin généraliste investigateur pourra recruter (=randomiser) et suivre jusqu'à 12 patients volontaires dans l'étude. Ce nombre est susceptible d'être modifié si des circonstances épidémiologiques exceptionnelles le justifient (concentration des cas sur une zone restreinte).

2 SCHEMA DE L'ETUDE

Contact et présentation de l'étude auprès des médecins libéraux investigateurs (réseau MEDOCEAN, environ 160 membres)

↓

Monitorage régional des cabinets (visite d'ouverture de centres)

↓

Information de l'investigateur de la période autorisée pour l'inclusion des patients
Présélection : Patient éligible et volontaire
Inclusion : consentement écrit et randomisation (250 patients)

↓

Contact avec le régulateur de l'étude pour envoi au domicile du patient inclus de l'infirmière-assistante de recherche clinique et du TEC
Prélèvement sanguin, stockage dans la glacière et envoi par coursier en fin de demi-journée au centre de virologie de l'étude (CHD ou GHSR). Réception et conditionnement (aliquotage des prélèvements) par personnel de laboratoire dédié.
Délivrance du traitement

Bras expérimental<br>chloroquine pendant 5 jours	Bras placebo<br>placebo pendant 5 jours
Auto-suivi de l'efficacité et la tolérance<br>deux fois par jour jusqu'à J5, puis chaque jour jusqu'à J14<br>Prélèvements sanguins à domicile (virémie, chloroquinémie J1, J3, J6 et J16)<br>Consultation à J7 et J25	

↓

Double saisie centralisée des données, réalisation des examens, stockage et envoi au laboratoire central de l'essai
Analyse et présentation des résultats en comité scientifique
Restitution des résultats auprès des médecins libéraux et des patients
Rapport final et publication scientifique des résultats de l'étude

FLOWCHART CURACHIK

Jour	1	3	5	6	7	14	16	25
**VISITE MEDECIN**	**X**				**X**			**X**
**VISITE INFIRMIERE**	**X**	**X**		**X**			**X**	
**Recueil du consentement**	X							
**Critères inclusion**	X							
**Randomisation**	X							
**Examen clinique**	X				X			X
**Formation remplissage de l'autoquestionnaire**	X							
**autoquestionnaire**	X	➔	➔	➔	➔	X		
**Traitements concomitants**	X				X			X
**Remise du thermomètre**	X							
**Dispensation traitement**	X							
**Prise traitement**	X	➔	X					
**Evénements indésirables**	X				X			X
**Prélèvement à domicile**								
**Bilan standard**	X						X	
**Sérologie (IgG/IgM)**	X			X			X	
**Chloroquinémie**	X	X		X			X	
**Charge virale**	X	X		X			X	
**Cytokines**		X						

3 INTRODUCTION ET JUSTIFICATION DE L'ETUDE

Une sévère épidémie de fièvre de Chikungunya a été observée sur l'île de la Réunion depuis le début de l'année 2006. L'agent étiologique est le virus Chikungunya, un arbovirus du genre *Alphavirus*, transmis par la piqûre de moustiques (sur l'île de la Réunion il s'agit du moustique *Aedes albopictus*).
A ce jour, plus de 210,000 cas ont été recensés. La plupart correspondent à des pathologies bénignes, avec fièvre et arthralgies intenses, mais également myalgies (91% des cas), céphalées (79%), éruption cutanée (52%), nausées ou vomissements (36%), symptômes respiratoires (31%), diarrhée (25%), hémorragies modérées (20%). Des cas plus sévères ont été recensés assez fréquemment avec en particulier une chronicisation des douleurs articulaires ou des rechutes hyperalgiques. A ce jour, des complications majeures (encéphalites, hépatites..) ayant nécessité une hospitalisation dans un service de réanimation ont été rapportées pour 85 cas de diagnostic confirmé. De plus, 34 cas d'infections néonatales ont été documentés. Enfin, 148 certificats de décès mentionnent le terme "Chikungunya" comme cause probable de la mort. Il est probable que les études de surmortalité qui seront menées à la fin de l'épidémie identifieront un nombre de décès en excès nettement supérieur à ce chiffre.
Au total, l'impact sanitaire mais également social et économique de cette épidémie est considérable et le pourcentage de personnes non-immunisées (environ 70% de la population) fait craindre de nouvelles poussées épidémiques dès le retour de l'été austral.

Il n'existe aujourd'hui aucun traitement antiviral contre le virus Chikungunya. Nous avons montré par des études *ex-vivo* (dans un modèle de cultures cellulaires susceptibles à l'infection virale) que la chloroquine avait un pouvoir inhibiteur significatif vis à vis de la réplication du virus Chikungunya. Cette efficacité est retrouvée à des doses correspondant aux doses plasmatiques observées chez des patients traités contre le paludisme avec cette molécule.

Nous proposons de tester l'efficacité de la chloroquine dans le traitement de l'infection humaine par le virus Chikungunya. Il s'agit d'un essai thérapeutique avec bénéfice individuel direct.

Justification du choix des doses de chloroquine
Le choix des doses est guidé par les données de la littérature dans 2 domaines différents:

- Traitement curatif par la nivaquine d'une maladie infectieuse au stade aigu

Le traitement curatif du paludisme à la phase aigue est parfaitement codifié chez l'adulte pour les souches chloroquino-sensibles. Il comporte une dose de charge de 2 fois 300 mg en une prise, suivie d'une dose de 300 mg 6 heures plus tard (dose totale le premier jour: 900 mg). Le traitement curatif est de 300 mg par jour en une prise.

- Traitement curatif par la nivaquine d'une maladie virale

Des essais *ex-vivo* portant sur l'inhibition de la réplication du virus Sindbis (un alphavirus proche du virus Chikungunya) ont montré une efficacité antivirale pour des doses de 3.2 à 32 µg/ml (J Virol. 1984;52:857-64. Cassell S et al.).
Nos propres essais ex-vivo (cultures cellulaires) portant sur l'inhibition de la réplication du virus Chikungunya (souche de la Réunion) ont par ailleurs montré une efficacité pour des doses de 1 à 10 µg/ml en fonction de l'inoculum viral:

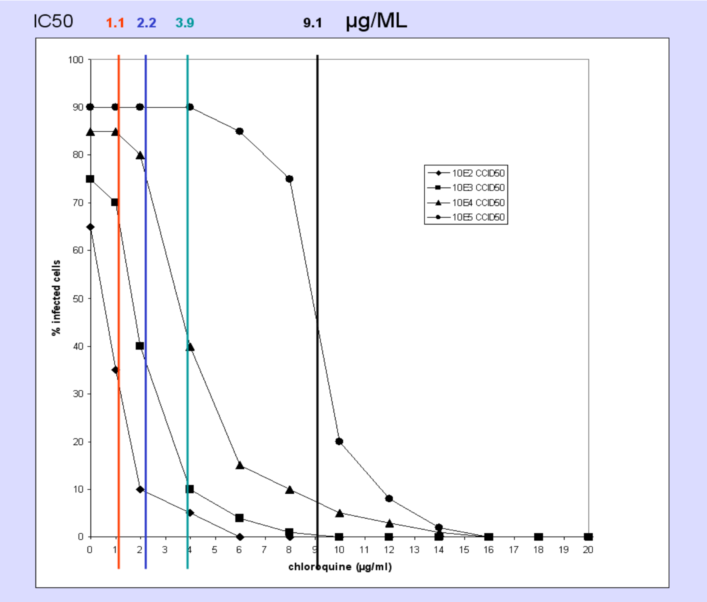

Des essais thérapeutiques pour le VIH ont été réalisés. Il a été montré qu'une dose de 2 fois 250 mg par jour en 2 prises était capable, en association avec la lamivudine et l'hydroxy-urée, de faire baisser la charge virale de plus de 1.5 logarithme de plus que le traitement par lamivudine seule ou hydroxy-urée seule (AIDS 2005; 19:1173-81. Lori F et al. ; J Assoc Phys India 2004; 52:597-98. Joshi SR et al. ; Lancet Infect Dis 2003; 3:722-27. Savarino A et al.). Des doses inférieures (équivalentes à deux fois 125 mg de chloroquine par jour) étaient inefficaces.

Au total, les données disponibles indiquent qu'il est important dans les infections aigues d'obtenir une chloroquinémie suffisante rapidement par une dose de charge. Dans le traitement d'entretien, les données sur le VIH suggèrent que les doses antivirales sont plus élevées que dans le traitement antiparasitaire (> 500 mg/jour).
Des doses soutenues sont également justifiées par le fait que l'efficacité antivirale est une fonction inverse de la charge virale (cf résultats expérimentaux) et que cette charge virale est particulièrement élevée dans le cas de l'infection par le virus chikungunya.

La durée du traitement est évaluée en fonction du mécanisme d'action attendu de la molécule *in vivo*, c'est-à-dire une activité de type antiviral. La virémie observée chez les patients de l'île de la Réunion dépassait rarement 5 jours après la date du diagnostic. La disparition de la virémie coïncide globalement avec l'apparition d'anticorps spécifiques de type IgM. L'activité antivirale de la chloroquine pourrait trouver son intérêt pendant la phase de réplication active du virus, et ne se justifie pas au-delà du cinquième jour, date moyenne de disparition de la virémie (un marqueur majeur de la réplication virale).

PROTOCOLE THERAPEUTIQUE RETENU:

Chloroquine, traitement de 5 jours *per os*:

- 600 mg le premier jour en une prise [soit 6 gélules de 100 mg] (J1)
- deux prises quotidiennes de 300 mg [soit 3 gélules de 100 mg] pendant 2 jours (J2 &J3)
- une prise quotidienne de 300 mg [soit 3 gélules de 100 mg] le soir pendant 2 jours (J4 & J5)

La dose globale reçue par le patient sera de 2400 mg. L'étalement de la prise médicamenteuse sur 5 jours, la détection par analyse biologique des patients insuffisants rénaux, l'exclusion des patients de moins de 40 kilogrammes de poids corporel, celle des patients atteints de maladies chroniques sévères et d'affections aiguës sont des garanties significatives pour prévenir les complications par surdosage.

4 OBJECTIFS DE L'ETUDE

4.1 Objectifs principal

Montrer une meilleure efficacité en termes de réduction de la durée des symptômes avec la chloroquine par rapport au placebo dans le traitement curatif du chikungunya clinique.

4.2 Objectifs secondaires

Montrer, pour la chloroquine par rapport au placebo seul :

- **une réduction de la charge virale et donc une réduction de la contagiosité (et possiblement des formes compliquées)**.
- Une réduction de la durée et la sévérité de chacun des symptômes suivis,
- Une réduction du nombre et de la durée des arrêts de travail attribuables au chikungunya,
- Une réduction de la fréquence de complications du chikungunya
- Une réduction de la consommation d'automédications (paracétamol, anti-inflammatoires, médications traditionnelles).

Comparer la tolérance au traitement dans les deux groupes

5 PLAN EXPERIMENTAL

5.1 Type de l'étude

Il s'agit d'un essai randomisé en double insu contre placebo en deux groupes parallèles.

5.2 Durée de l'étude

Pour chaque sujet la durée du traitement sera de 5 jours. Le sujet sera suivi par son médecin traitant qui le verra à l'inclusion (J1), à J7 et en fin de protocole (J25). Le sujet effectuera un auto-suivi de ses symptômes pendant une durée de 14 jours. Il rapportera son observance thérapeutique durant les 5 jours de traitement. La durée maximale de participation à l'étude d'un sujet sera de 25+/- 2 jours.

5.3 Nombre prévu de sujets

A partir des patients éligibles, il est prévu de randomiser 250 patients ayant un diagnostic clinique typique de chikungunya, soit 125 patients dans chaque bras de l'essai. Le monitorage permettra de piloter le rythme et la répartition des inclusions en temps réel et d'ajuster éventuellement la demande d'inclusions auprès des médecins en contact avec un plus grand nombre de cas. Chaque médecin investigateur ne pourra cependant pas inclure (= randomiser) plus de 12 sujets dans l'étude. La justification statistique de l'effectif nécessaire est donnée au § 6.3.

5.4 Critères d'éligibilité pour une éventuelle inclusion

Patient volontaire pour participer à l'essai, n'ayant pas présenté de chikungunya connu dans ses antécédents et paraissant capable de comprendre et de remplir le carnet d'autoévaluation qui lui sera remis à l'issue de la consultation (ou de le faire remplir par un proche, sur ses indications).

Patient de 18 ans à 65 ans, de sexe masculin ou féminin, ayant donné son accord écrit de participation à l'essai.

Patient ayant un poids corporel supérieur ou égal à 60 kilogrammes.

Patient présentant des arthralgies d'apparition brutale dans un contexte fébrile (fièvre supérieure à 38°C).

Patient ayant un début de sa symptomatologie datant de moins de 48 heures.

Patient ayant accès à un téléphone.

Patient susceptible d'être suivi pendant toute la durée de l'essai.

5.5 Critères d'inclusion

Est éligible pour la randomisation un patient ayant rempli tous les critères d'inclusion et ayant donné son consentement éclairé écrit.

5.6 Critères de non-inclusion

Patient ne remplissant pas l'ensemble des critères d'inclusion.
Patient de plus de 66 ans.
Patient de moins de 60 kilogrammes
Patient présentant une contre-indication pour la chloroquine, en particulier une hypersensibilité à la chloroquine, à ses dérivés ou à l'un des constituants
Patient ayant pris de la chloroquine dans les 90 jours qui précèdent.
Femme en âge de procréer sans contraception en cours.
Femme enceinte ou allaitante.
Patient connu pour une insuffisance rénale (clairance à la créatinine de moins de 60 ml/mn ou créatininémie >120) ou pour une rétinopathie.
Patient présentant une maladie cœliaque ou une porphyrie
Patient avec une maladie intercurrente grave non équilibrée, une maladie hépatique sévére, une maladie coeliaque.

Remarque:

Les femmes enceintes et les femmes en âge de procréer sans contraception en cours sont *a priori* exclues de l'étude. Pour toutes les femmes entrant dans l'étude, un test de grossesse sera réalisé par précaution sur le sérum prélevé à J1. Sa positivité entraînera l'arrêt du traitement par la chloroquine et l'exclusion de la patiente de la suite de l'étude.
L'inclusion sera effectuée sans attendre le résultat du dosage de la créatininémie. Une valeur >120 entraînera l'arrêt du traitement par la chloroquine et l'exclusion du patient de la suite de l'étude.

Les dosages biologiques (test de grossesse, créatininémie) seront réalisés dans les 12 heures suivant le prélèvement sanguin et le résultat sera communiqué dans un délai de 18 heures au médecin investigateur. La prise médicamenteuse sera stoppée dans tous les cas (au plus tard) après la prise matinale à J2.

5.7 Evaluation pré-thérapeutique

Elle sera réalisée par le médecin traitant. Lorsque le patient rassemblera l'ensemble des critères d'inclusion, le patient procédera en présence du TEC à une prise de température buccale avec un thermomètre fourni, puis l'infirmière-assistante de recherche clinique pratiquera un prélèvement sanguin avant l'administration de la première dose de traitement (chloroquine ou placebo en aveugle: 600 mg en une prise).

5.8 Visite d'entrée

5.8.1 Evaluation clinique

Le médecin traitant investigateur remplira un cahier d'observation (voir annexe) dont la première page regroupera l'ensemble des critères d'inclusion. Ne seront randomisés par le médecin que les patients volontaires remplissant l'ensemble des critères d'éligibilité pour l'inclusion.

5.8.2 Evaluation biologique

A J1, un prélèvements sanguin (bilan standard [FNS-plaquettes, ionogramme-urée-créatinine-glycémie, transaminases-γGT-phosphatases alcalines-bilirubine, CRP] + sérologie IgG/IgM + charge virale + isolement viral + chloroquinémie).
A J3, un prélèvement sanguin (charge virale + chloroquinémie + cytokines).
A J6, un prélèvement sanguin (sérologie IgG/IgM + charge virale + chloroquinémie).
A J16, un prélèvement sanguin (bilan standard [FNS-plaquettes, ionogramme-urée-créatinine-glycémie, transaminases-γGT-phosphatases alcalines-bilirubine, CRP] + sérologie IgG/IgM + charge virale + chloroquinémie).

Les prélèvements seront acheminés par coursier après stockage en glacière par le binôme de monitorage mobile au centre hospitalier régional de l'essai.

5.9 Randomisation et désignation du numéro des sujets

Une liste de randomisation sera fournie à chaque médecin généraliste investigateur. La randomisation est stratifiée par binôme qui administrera aux patients les produits selon un ordre croissant.
Les traitements seront pré-randomisés avant l'envoi dans les pharmacies hospitalières et les TEC auront à donner les lots qui seront distribués par la pharmacie hospitalière qui devra respecter l'ordre croissant, au fur et à mesure des inclusions.

5.10 Traitements étudiés

Bras expérimental : Chloroquine, 600 mg en une prise le premier jour; puis 300 mg, 2 fois/j, *per os* pendant 2 jours, soit matin et soir; puis 300 mg, 1 fois/j le soir, *per os* pendant 2 jours.

Bras de référence : Placebo, 600 mg en une prise le premier jour; puis 300 mg, 2 fois/j, *per os* pendant 2 jours, soit matin et soir; puis 300 mg, 1 fois/j le soir, *per os* pendant 2 jours.

En cas de problème de tolérance, le traitement pourra être interrompu.

Dispensation des médicaments et des kits de prélèvements étudiés
Les pharmacies hospitalières des centres hospitaliers régionaux participant à l'essai réceptionnent les médicaments de l'essai fournis par le laboratoire Sanofi-Aventis et les transmettent aux TECs régionaux pour distribution de lots aux patients randomisés par les médecins après la prescription médicale rédigée lors de la visite d'inclusion.
Les médecins, les TEC et patients restent à l'insu du traitement attribué jusqu'à la fin de l'essai.
Le traitement est transmis au patient par le TEC qui lui fournit ainsi les explications nécessaires. La première prise est effectuée en présence du TEC qui consigne l'heure d'administration.

5.11 Administration des médicaments étudiés

Première prise à J1 en présence du TEC. Auto-administration par les patients pendant 5 jours. Les médecins généralistes peuvent être amenés à prescrire du paracétamol ou un anti-inflammatoire à leurs patients ; ils restent libres de leurs prescriptions par ailleurs. Les patients peuvent être amenés à s'auto-médiquer. Tous ces traitements associés sont notés dans le cahier d'observation.
Un traitement symptomatique de référence sera proposé aux généralistes qui resteront libres de leur choix thérapeutique.

5.12 Suivi des patients et recueil des données

5.12.1 Suivi des patients

Le suivi est assuré par les médecins généralistes investigateurs pour la partie clinique, par des consultations à J1 (consultation de présélection, signes cliniques à l'exception des critères principaux de jugement et de ceux donnant lieu à quantification par auto-questionnaire remis au patient, consentement écrit du patient, randomisation), J7 (signes cliniques à l'exception des critères principaux de jugement et de ceux donnant lieu à quantification par auto-questionnaire remis au patient, observance, événements indésirables) et J25 (signes cliniques, observance, événements indésirables).
Le suivi est également assuré par les infirmières et TECs lors de leurs visites (J3, J6, J16), en particulier pour s'assurer du recueil des données par autoquestionnaire et répondre aux questions pratiques éventuelles.

5.12.2 Recueil des données

Les médecins généralistes assurent le recueil des données cliniques sur un document spécifique (Annexe) lors des consultations à J1, J7 et J25.

Les TECs de l'essai récupèrent le document d'autoévaluation à J16.

Les patients consignent les données d'auto-évaluation et le recueil de la température corporelle en présence du TEC à J1, 2 fois par jour jusqu'à J5, puis 1 fois par jour jusqu'à J14 sur le carnet qui leur est remis à cet effet (Annexe).

Les enquêteurs vérifient la tenue du carnet, notamment des éléments du carnet patient journalier et la prise de la température (qui participe au carnet patient journalier) lors des visites à J1, J3, J6 et J16. Ils récupèrent le document d'autoévaluation à J16.

La double saisie des cahiers d'observation anonymisés est sous-traitée par un prestataire professionnel et répond aux exigences nationales en matière d'informatique et de protection des libertés.

5.12.3 Réseaux de médecins libéraux

Les médecins généralistes du comité scientifique de l'essai sont chargés d'identifier dans chacune des communes où se déroule l'essai, des médecins généralistes investigateurs volontaires pour y participer, en premier lieu ceux du réseau Medocéan. L'objectif est de sélectionner dans l'essai (et de randomiser) les 250 premiers patients répondant aux critères d'inclusion. Ainsi 50 médecins généralistes pourraient être amenés à randomiser au maximum 12 patients dans les deux bras de l'essai. Il sera proposé aux médecins de randomiser un nombre pair de patients.

5.12.4 Information des sujets

Ce sont les médecins généralistes traitants eux-mêmes, investigateurs de cet essai, qui procèderont au recueil du consentement éclairé écrit des patients éligibles.
Le formulaire de consentement sera remis au patient qui disposera du temps suffisant pour lire la note d'information et signer son consentement. Cet accord sera signé avant la randomisation, la prise de sang, l'administration de la 1ère dose de traitement de l'essai, la remise de la suite du traitement et la détermination des autres rendez-vous prévus.

5.12.5 Liste des données recueillies

Recueil à J1 par le médecin généraliste investigateur
- Visite de sélection (vérification des critères d'éligibilité, d'inclusion et de non inclusion)
- Vérification de l'obtention du consentement éclairé écrit
- Date de la visite, âge du patient, antécédents de maladie de chikungunya, sexe.
- Date d'apparition et nature des premiers symptômes ;
- Date d'apparition de la fièvre supérieure à 38°C et de l'arthralgie ;

- signes cliniques à l'exception des critères principaux de jugement et de ceux donnant lieu à quantification par auto-questionnaire remis au patient: intensité des symptômes cliniques sur une échelle cotée de 0 à 3 (cf Annexe) ;

Recueil à J1 par le TEC
- Aide au recueil des éléments d'autoévaluation par le patient et de la température.
- Réalisation, conditionnement et envoi du prélèvement destiné au laboratoire de virologie (qui assure la distribution aux laboratoires compétents).
- Recueil de l'heure de la première prise du traitement (la première prise est administrée en présence du TEC).

Recueil à J3 par l'infirmière
L'infirmière recherchera les signes suivants pouvant témoigner d'une éventuelle intolérance à la chloroquine : désorientation temporo-spatiale (réponse à une interrogation simple sur la date et le lieu d'examen), troubles de la vue, vomissements, éruption cutanée, étourdissements.
La présence d'un de ces signes (constaté ou rapporté) ou de tout signe clinique inhabituel entraînera un signalement immédiat au médecin investigateur qui décidera de l'opportunité de l'arrêt du traitement.

Recueil à J7 et J25 par le médecin généraliste investigateur
- signes cliniques (voir Annexe). Date de la visite.
- événements indésirables rapportés et/ou constatés.

Recueil J1 à J14 par le patient : autoquestionnaire (carnet) remis au patient par le TEC et rendu à ce dernier à J16
- **Température** par thermomètre digital fourni au patient à J1 (par voie buccale)
- Auto-évaluation des signes cliniques par le patient deux fois par jour pendant les 5 jours de traitement
- **Arthralgies:** localisation et intensité sur une échelle cotée 0 (= absent), 1 (= minime), 2 (= modérée), 3 (= important).
- asthénie, céphalée, myalgies sur une échelle cotée 0 à 3 ;
- recueil sur une échelle visuelle analogique validée de 10 points des capacités à effectuer des activités normales, de l'état général, de la qualité du sommeil.
- Recueil sur le carnet des prises de médicaments (ceux de l'essai, d'autres prescrits, d'autres en automédication)
- événements indésirables auto-rapportés par le patient
- recueil de l'observance rapportée (auto-évaluée avec la grille APROCO, Carrieri et al. JAIDS 2001 ;28 :232-39)

Recueil de J1, J3, J6 et J16 pour les examens virologiques
Réalisation du prélèvement selon le mode opératoire fourni en annexe et conservation à +4°C pendant moins de six heures, puis envoi par coursier en glacière aux laboratoires des centres hospitaliers régionaux compétents.

5.12.6 Randomisation et mise en œuvre des traitements

Après vérification de l'ensemble des critères d'inclusion, le médecin généraliste traitant investigateur recueille le consentement éclairé écrit du patient. En fonction du groupe de randomisation, le TEC en insu attribue un traitement A ou B et remet les échantillons de médicaments au patient selon la prescription médicale en lui expliquant les modalités d'auto-administration, pour une durée de 5 jours.

6 STATISTIQUES

6.1 Variables d'analyse

- Données recueillies par le médecin généraliste investigateur à J1 : âge du patient, antécédent de chikungunya dans les 12 derniers mois, sexe, signes cliniques à l'exception des critères principaux de jugement et de ceux donnant lieu à quantification par auto-questionnaire remis au patient: intensité des symptômes cliniques sur une échelle cotée de 0 à 3 ; date d'apparition des premiers signes.

- Auto-suivi des symptômes cliniques à J1 avec l'aide du TEC, puis deux fois par jour entre J1 et J5, et tous les jours jusqu'à J14 :

  - **température** matinale et vespérale ;
  - **arthralgies** : localisation et intensité des symptômes sur une échelle cotée de 0 à 3 ;
  - asthénie, céphalée, myalgies : intensité des symptômes sur une échelle cotée de 0 à 3 ;
  - capacités à effectuer des activités normales, de l'état général, de la qualité du sommeil: cotées sur échelle visuelle analogique validée de 10 points ;
  - autres prises médicamenteuses.

- Résultats des examens virologiques à J1, J3, J6 et J16.
  - RT-PCR quantitative pour le génome du virus chikungunya (J1, J3, J6 et J16)
  - Isolement en culture déterminé par test d'immunofluorescence sur culture cellulaire (cellules Vero E6) sur tous prélèvements positifs en RT-PCR
  - Chloroquinémie (chez tous les patients traités ou placébo) (J1, J3, J6 et J16)
  - Marqueurs de la réponse immunitaire, dont sérologies IgG/IgM anti-chikungunya (J1, J6 et J16) et dosages de cytokines (J3).

- Données du bilan biologique standard (J1 et J16)
  - NFS-plaquettes,
  - Ionogramme, urée, créatinine, glycémie, transaminases, γGT, phosphatases alcalines, bilirubine, CRP

- Données recueillies par le médecin généraliste investigateur à J7: signes cliniques à l'exception des critères principaux de jugement et de ceux donnant lieu à quantification par auto-questionnaire remis au patient: intensité des symptômes cliniques sur une échelle cotée de 0 à 3 [(signes dermatologiques (éruption, pigmentation), digestifs (nausées, vomissements, diarrhée), respiratoires, hémorragiques)] et J25: température, arthralgies, céphalée, asthénie, myalgies, frissons, autres signes cliniques (signes dermatologiques (éruption, pigmentation), digestifs (nausées, vomissements, diarrhée), respiratoires, hémorragiques) : intensité des symptômes cliniques sur une échelle cotée de 0 à 3.

- Observance rapportée par le patient. Evénements indésirables rapportés par le médecin et par le patient.

CONSTRUCTION DU CRITERE DE JUGEMENT PRINCIPAL CLINIQUE:
"amélioration des 2 symptômes cardinaux de la maladie (température et arthralgies spontanées)", définie comme :

- la disparition de la fièvre (T° inférieure à 38°C)
- ET le passage d'un stade 3 (important) ou 2 (modéré) à un stade 1 (minime) ou 0 (absent) pour les arthralgies
- ET pour une durée supérieure à 24 heures pour ces 2 symptômes.

CONSTRUCTION DU CRITERE DE JUGEMENT SECONDAIRE BIOLOGIQUE:
"diminution de la virémie", définie comme :

- raccourcissement de la durée de virémie détectable
- OU diminution de la charge virale à J3.

6.2 Méthodes d'analyse statistique

Les durées de symptômes seront comparées entre les deux groupes de randomisation par un test de Mantel-Haenszel stratifié par binôme. Les autres variables continues seront comparées par un test de Wilcoxon modifié pour tenir compte de la stratification. Les variables qualitatives seront analysées par des tests de Mantel-Haenszel stratifiés. Les tests seront effectués en formulation bilatérale, au seuil alpha de 5 %. Le logiciel SAS, version 7 ou ultérieure, ou le logiciel R sera utilisé pour réalisé les analyses dans le centre de données de l'Inserm-UPMC UMR-S707

6.2.1 Comparabilité des groupes

La comparabilité des groupes sera vérifiée pour l'ensemble des variables recueillies par le médecin et auto-recueillies par le patient à J1, ainsi que pour les données virologiques.

6.2.2 Analyse des critères principaux

Chaque critère de jugement principal est comparé entre les deux bras de randomisation selon deux analyses statistiques. **L'analyse principale est l'analyse en intention de traiter (ITT) du critère principal** portant sur les patients ayant au moins reçu une dose de traitement. Les résultats portant sur

la différence des durées des symptômes seront présentées selon la médiane, la fourchette (minimum et maximum), et/ou l'intervalle de confiance à 95 %.
Une seconde analyse sera réalisée chez tous les patients ayant au moins reçu une dose de traitement et ayant un résultat de culture cellulaire positive pour un virus chikungunya ou un résultat de RT-PCR positive pour un virus chikungunya. Une troisième et une quatrième **analyse « per protocole »** porteront respectivement sur les mêmes populations de l'essai que définies ci-dessous, mais sur la **"diminution de la virémie"**, définie comme :

- raccourcissement de la durée de virémie détectable
- OU diminution de la charge virale à J3.

6.2.3 Analyse des critères secondaires

Les mêmes méthodes d'analyses seront appliquées pour les autres critères de jugement de l'essai. Les événements indésirables seront présentés dans des tableaux comparatifs où seules les différences significatives seront signalées.

Critères secondaires:

- la durée d'amélioration de chacun des critères cliniques non cardinaux pris séparément (myalgies, céphalée, autres signes). L'amélioration est définie comme le passage d'un degré 2 ou 3 à un degré 0 ou 1 sur l'échelle d'auto-évaluation en 4 degrés (0, 1, 2, 3).

- la durée d'amélioration des signes cardinaux depuis le début de la maladie
- la durée d'amélioration de tous les critères cliniques

- la durée de disparition des signes cardinaux
- la durée de disparition de chacun des critères cliniques pris séparément
- la durée de disparition de tous les critères cliniques

- la durée de retour à l'activité normale
- le nombre et la durée des arrêts maladie (pour les patients ayant une activité professionnelle)
- les rechutes hyperalgiques

- la consommation d'antalgiques, AINS, médications traditionnelles
- la présence d'une complication
- la nécessité d'une hospitalisation pour complication
- les effets secondaires graves et les décès

6.2.4 Analyse médico-économique

L'ensemble des événements de santé intervenant au cours ou au décours de l'épisode de chikungunya seront notés sur la période de 5 jours (consommation d'autres médicaments à visée symptomatique, prescriptions médicamenteuses, arrêt de travail, nouvelle consultation, hospitalisation). Ces événements seront valorisés selon la nomenclature de la sécurité sociale et les coûts du point de vue de l'assurance maladie seront chiffrés et comparés entre les groupes de traitement après 5 jours d'inclusion. Ils seront rapportés aux bénéfices observés éventuellement avec le traitement expérimental.

6.3 Justification du nombre de sujets

Un effectif de 100 sujets par bras de randomisation, soit au total 200 sujets remplissant les critères d'inclusion, sera nécessaire à constituer pour mettre en évidence une différence entre le bras expérimental (chloroquine) par rapport au bras témoin (placebo) de deux jours sur la durée des symptômes (pour un écart-type de 2), avec une puissance de 80 %, pour un test en formulation bilatérale avec un seuil de signification $P < 0{,}05$.
Le risque de diagnostic clinique erroné (faux positifs) pouvant dans les premières études réalisée à la Réunion avoisiner 20%, l'effectif final a été fixé à 125 sujets par bras, soit 250 au total.

7 JUSTIFICATION DU RECOURS A DES DONNEES INDIRECTEMENT NOMINATIVES

Le recueil et le traitement informatique de ces informations entrent dans le cadre des lois n°78-17 du 6 janvier 1978 et n°94-548 du 1er juillet 1994 (dites "Informatique et Libertés"). Les patients reçoivent une information détaillée quant à la nature des données recueillies, leur utilisation prévue, l'objectif de

l'essai, les destinataires des données, le droit d'accès et de rectification, ainsi que le droit d'opposition ou d'arrêt de l'essai sans justification à fournir.
Seuls les patients éligibles ayant accepté de donner leur consentement exprès écrit et éclairé préalable, après avoir pu lire une lettre d'information qui leur a été expliquée sont inclus.

8 JUSTIFICATION DE L'APPLICATION DE LA LOI HURIET

L'étude s'inscrit dans le champ d'application des articles L.209-1 et suivants du Code de la Santé Publique de la Loi dite " loi Huriet " du fait de l'intervention ou de la modification de la prise en charge médicale habituelle des sujets.

9 SURVEILLANCE DES EVENEMENTS INDESIRABLES

9.1 Surveillance des événements indésirables et des événements indésirables graves

Tous les événements seront pris en charge et signalés conformément à l'ensemble des réglementations applicables et seront mentionnés dans le rapport final de l'étude clinique.

9.2 Définition des événements indésirables (EI) et des événements indésirables graves (EIG)

Un **événement indésirable** est un signe, symptôme, syndrome, une maladie ou une anomalie biologique survenant chez un patient ou un sujet participant à une étude clinique recevant un médicament. Ce terme n'implique pas un lien de causalité avec le traitement en question.

On entend par événement indésirable :

Toute manifestation nocive à caractère médical, associée ou observée à l'occasion de l'utilisation d'un médicament ou produit biologique (à quelle que dose que ce soit) ou d'un dispositif médical, survenant chez un patient ou chez un sujet participant à une étude clinique, que cette manifestation soit considérée ou non comme liée à ce produit.

Un événement indésirable peut donc être une manifestation non voulue et défavorable (telle que par exemple un paramètre biologique anormal), un symptôme ou une maladie, associé de façon temporelle à l'utilisation d'un produit, que cet événement soit ou non lié à celui-ci.

Ces évènements indésirables regroupent les manifestations :
- Survenant lors de l'utilisation d'un de ces produits,
- Associées ou observées lors d'un surdosage accidentel ou volontaire,
- Associées ou observées lors d'un abus de produit,
- Associées ou observées lors d'un sevrage ou d'un arrêt du produit.

Si un événement indésirable est détecté durant la période de l'étude clinique, il doit être reporté dans le cahier d'observation, qu'il soit modéré, sévère ou discret.
On entend par événement indésirable grave, tout événement indésirable ayant une des conséquences suivantes :
- décès,
- mis en jeu du pronostic vital (risque immédiat de décès),
- hospitalisation ou prolongation d'hospitalisation,
- incapacité ou invalidité, temporaire, persistante ou significative
- anomalie ou malformation congénitale, cancer.

Remarque : « Mettre en jeu le pronostic vital » signifie que le sujet était en danger immédiat de mort lors de la survenue de l'événement. Ce cas ne correspond pas à un événement qui aurait pu hypothétiquement entraîner la mort s'il avait été plus sévère ».

Sont également considérés comme graves, les évènements médicalement importants (n'ayant pas entraîné le décès ou mis en jeu le pronostic mais qui, après jugement médical approprié, peuvent mettre en danger le patient ou le sujet et peuvent nécessiter une intervention médicale ou chirurgicale pour prévenir une des conséquences citées plus haut).
Ex : bronchospasme allergique, pharmacodépendance, abus…

La relation de causalité entre chaque événement indésirable et le médicament doit être évalué :

- improbable
  - chronologie non évocatrice,
  - ou étiologie de l'événement identifié,
  - ou médicament ne pouvant être impliqué
- possible
  - chronologie évocatrice mais d'autres étiologies peuvent être la cause. Cependant, l'implication du médicament ne peut pas être exclue (documenté).
- probable
  - chronologie évocatrice, d'autres étiologies sont possibles mais improbables (documenté).
  - Le caractère attendu ou inattendu de l'événement indésirable grave sera défini en fonction des effets indésirables retrouvés dans le résumé des caractéristiques du produit.

L'investigateur sera seul juge des décisions à prendre concernant la sécurité du sujet en cas d'effet secondaire.

Pour les évènements indésirables graves, notamment pour les décès, le délai de déclaration au promoteur est : immédiatement après que l'investigateur a eu connaissance de l'événement par fax, ou par téléphone obligatoirement suivi d'un fax au numéro suivant 04.91.38.14.79. (DRC Marseille).

En effet, le promoteur doit garder des traces de tout événement indésirable grave et doit prendre connaissance avec les investigateurs de tout effet secondaire et si nécessaire le rapporter aux autorités sanitaires.
L'ensemble des centres participant à l'étude doit être informé de tout événement indésirable grave sous les 10 jours.

Les cas de grossesse et de surdosages devront aussi donner lieu à une déclaration.

9.3 Obligations de l'investigateur en matière de déclaration des événements indésirables

Événements indésirables

Les événements indésirables, quels qu'en soient la gravité et le lien causal avec le produit à l'étude, survenant entre la première visite prévue par le protocole de l'essai clinique/la signature du formulaire de consentement éclairé et la dernière visite prévue par le protocole, doivent tous être consignés sur les pages correspondantes du cahier d'observation. Lorsque cela est possible, les symptômes devront être regroupés au sein d'un seul et même syndrome ou diagnostic. L'investigateur devra préciser la date de survenue de l'événement, son intensité, les mesures prises s'agissant du produit à l'étude, le traitement entrepris, l'évolution finale et son avis sur la relation causale entre l'événement indésirable et le produit à l'étude.

Les anomalies des analyses de laboratoire, des signes vitaux ou de l'ECG doivent être rapportées comme des événements indésirables uniquement si elles présentent une pertinence médicale : symptomatiques, ou nécessitant un traitement, ou aboutissant à l'arrêt de l'étude et/ou répondant à un critère de gravité.

Événements indésirables graves

En cas d'événement indésirable grave, l'investigateur doit immédiatement :

- ENVOYER (dans les 24 heures ouvrables) les pages correspondantes du cahier d'observation, signées et datées, au représentant de l'équipe de monitoring dont le nom, l'adresse et le numéro de fax sont inscrits dans le protocole de l'essai clinique ;

- JOINDRE la photocopie de tous les examens effectués et indiquer les dates auxquelles ceux-ci ont été réalisés. Il convient de préserver l'anonymat du patient et de mentionner les identifiants du patient dans le cadre de l'essai clinique sur toute copie de documents originaux communiquée au promoteur de l'étude. S'agissant des résultats d'analyses, mentionner les normes du laboratoire.

- Les informations sur le suivi des événements indésirables graves ayant entraîné le décès ou mettant en danger le pronostic vital doivent être communiquées dans un délai supplémentaire d'une semaine calendaire.

Suivi

- L'investigateur doit prendre toutes les mesures appropriées pour veiller à la sécurité des patients, et notamment assurer le suivi de l'évolution de tout événement indésirable (signes cliniques, résultats d'analyses ou autres) jusqu'au retour à la normale ou jusqu'à la consolidation de l'état du patient.
- En cas d'événement indésirable grave, le patient doit être suivi jusqu'à son rétablissement et jusqu'à ce que les résultats d'analyses soient revenus à la normale, ou jusqu'à ce que la progression ait pu être stabilisée. Ceci implique que le suivi pourra être prolongé au-delà de la participation du patient à l'essai clinique et que des informations complémentaires pourront être demandées par l'équipe de monitoring.
- Si un événement indésirable grave est porté à l'attention de l'investigateur à tout moment après l'arrêt de la prise du produit à l'étude et si l'investigateur considère que cet événement pourrait raisonnablement avoir été causé par le produit à l'étude, celui-ci devra être signalé à l'équipe de monitoring.

9.4 Grossesse

- En cas de grossesse, la prise du produit à l'étude devra être interrompue et le promoteur de l'étude devra en être informé **immédiatement**.
- Le suivi de la grossesse devra obligatoirement être assuré jusqu'à ce que l'issue ait pu en être déterminée.
- La grossesse sera consignée comme un EI dans tous les cas. Elle sera décrite comme un EIG uniquement si elle remplit les critères des EIG.

9.5 Obligations du promoteur

Au cours de l'étude, le promoteur signalera sans délai aux investigateurs, aux autorités et au Comité de Protection des Personnes tous les EIG à la fois inattendus et raisonnablement liés au produit étudié.

Tout EIG non répertorié parmi les événements prévus dans le RCP en vigueur en France sera considéré comme inattendu.

Le promoteur de l'étude consignera tous les événements indésirables recueillis pendant le déroulement de l'étude au sein du rapport d'essai clinique (REC).

10 CONSIDERATIONS ETHIQUES ET LEGALES

10.1 Cadre réglementaire de l'étude

Le protocole de l'étude modifie la prise en charge médicale habituelle du sujet.
Les procédures décrites en annexe de ce protocole, concernant la conduite, l'évaluation et la documentation de l'étude sont conçues pour assurer que les différents partenaires de l'essai et les investigateurs respectent les Bonnes Pratiques Cliniques et les principes éthiques définis dans la Déclaration d'Helsinki.
L'étude sera conduite conformément à la Loi informatique et libertés du 6 Janvier 1978 modifiée par la loi du 1er Juillet 1994 et complétée par la loi du 6 août 2004.

10.2 Obligations légales

Cette étude sera soumise à l'autorisation de la Commission Nationale de l'Informatique et des Libertés.
Avant toute analyse impliquant une manipulation de données identifiantes, toutes les obligations éthiques et légales seront respectées.
Le promoteur conservera une trace de toutes les communications avec les autorités.

10.3 Information des sujets

Tous les traitements sont réalisés sur des données non identifiantes.

10.4 Protection des données personnelles

Les données des sujets seront recueillies en accord avec la Loi Informatique et Libertés du 6 Janvier 1978 modifiée par la Loi du 1er Juillet 1994 et la loi du 6 août 2004.
Aucune donnée nominative, directement ou indirectement nominative, ne sera transmise à quiconque par le médecin traitant le patient. Seules des données anonymes et résumées seront communiquées par l'investigateur médecin généraliste traitant dans le cadre de l'analyse statistique et de la publication des résultats.

10.5 Comité indépendant de suivi de l'essai

Le promoteur met en place un comité indépendant de suivi de l'essai. Il a la possibilité à tout moment de proposer une réorientation de l'essai en fonction de l'évolution de la situation épidémique (ou pandémique). Il intervient aussi, à la demande du promoteur, en cas de question de sécurité ou d'éthique survenant au cours de l'essai.

10.6 Arrêt prématuré de l'étude

Le promoteur pourra interrompre l'étude à tout moment. Cette interruption devra avoir lieu si possible après concertation mutuelle avec les partenaires de l'étude et après consultation du comité indépendant de suivi de l'essai.

11 MONITORAGE ET CONTROLE DES DONNEES

Le monitorage sera effectué sous la direction de la cellule Contrôle Qualité de l’Assistance Publique-Hôpitaux de Marseille selon les procédures en vigueur dans l’établissement.
L’AP-HM pourra déléguer pour partie ce monitorage au CIC de l’Inserm-La Réunion.
Les attachés de recherche clinique du CIC de l’Inserm devront se conformer aux procédures de contrôle qualité appliquées à la recherche clinique du promoteur.

Les investigateurs acceptent en signant le protocole l’application de ces procédures d’audit et l’ouverture des dossiers médicaux des patients inclus dans l’essai, dans le cadre des audits internes et externes menés dans le cadre de cet essai par les personnes qualifiées par le promoteur et/ou les autorités de santé, dans le respect de la réglementation en vigueur.

12 DOCUMENTATION ET UTILISATION DES RESULTATS DE L’ETUDE

12.1 Documentation des résultats de l’étude

Toutes les données recueillies dans le cadre du protocole seront saisies dans le cahier d'observation par l’investigateur ou le TEC. Les modalités de remplissage et de corrections des cahiers d'observation seront expliquées à l’investigateur.
L’investigateur devra remplir le cahier d'observation au moment même des visites ou consultations. Il faudra fournir une explication pour toutes les données manquantes.
Une fois clos, le cahier d'observation devra être revu et validé par l'Attaché de Recherche Clinique régional ou par un personnel désigné par le promoteur.

Tous les cahiers d'observation seront conservés par le promoteur.

12.2 Utilisation des résultats de l’étude

Le promoteur fournit un cahier d'observation par sujet.
Toutes les données recueillies dans le cadre du protocole devront être saisies dans le cahier d'observation par l'investigateur ou le TEC. Les modalités de saisie et de modification des cahiers d'observation seront expliquées à l'investigateur. Si l'investigateur délègue à certaines personnes le droit de saisir des données dans le cahier d'observation, il devra fournir au promoteur la liste des personnes autorisées avec leurs noms, fonctions, identification.
L'investigateur ou le représentant désigné devra saisir le cahier d'observation le plus tôt possible, après le recueil de l'information, de préférence le jour même de la visite ou de la consultation. Il faudra fournir une explication pour toutes les données manquantes.
Les auto-questionnaires des patients remis au médecin généraliste investigateur feront l’objet d’une double saisie organisée par le promoteur.
Tous les cahiers d'observation seront conservés par le promoteur. L'investigateur conservera une impression papier de tous les cahiers d'observation remplis.
Toutes les informations relatives à cet essai, ou les données scientifiques fournies par le promoteur et non encore publiées, sont confidentielles. Leur propriété est réglée par un contrat entre le promoteur et Sanofi-Aventis.
Les cahiers d'observation saisis dans le cadre de l'étude sont la propriété à part entière du promoteur.
Le promoteur rédigera un rapport final de l'étude. Le rapport final de l’étude sera remis (pour information) aux partenaires de l’étude.
Les résultats de l’étude feront l’objet d’une restitution auprès des médecins investigateurs participants et aux patients sous une forme appropriée.
Les résultats de l’étude feront l’objet de publications dans des journaux scientifiques et de présentations lors de réunions scientifiques. Pour toute publication, les investigateurs principaux élaboreront à l’avance avec l’ensemble des investigateurs du Conseil Scientifique des règles de valorisation dont ils assureront l’arbitrage.

13 DUREE DE L'ETUDE ET CALENDRIER

- Les inclusions se dérouleront en mai 2006 dès l'obtention des accords réglementaires, si les conditions épidémiologiques le permettent.
- Les données seront saisies en double saisie par une société prestataire de service.
- L'analyse et le rapport final seront remis en juillet 2006.

Si les financements et/ou les accords réglementaires n'étaient pas obtenus en temps utile, l'étude serait repoussée jusqu'à l'obtention de ces derniers.

14 ANNEXES

14.1 Engagements du promoteur et de l'investigateur

Le protocole a été revu et approuvé par le comité scientifique de l'essai et le sera par le promoteur avant sa mise en oeuvre. L'information contenue est conforme aux principes moraux, éthiques et scientifiques définis dans la déclaration d'Helsinki, et dans les lois et réglementations françaises.

Les investigateurs seront informés de toutes les données nouvelles ou significatives, de tout élément nouveau concernant l'étude.

14.2 Cahier d'observation

14.2.1 Informations recueillies par le médecin généraliste investigateur

Le cahier d'observation est joint en annexe ; il suit les recommandations de la CNIL.
Visite ou consultation de présélection à J1

- initiales du nom et du prénom, sexe, date de naissance
- critères d'inclusion

Inclusion et randomisation

- prélèvement

Visite ou consultation à J7 et J25

- signes cliniques à J7 et J25
- recueil des événements indésirables

14.2.2 Données du bilan standard et du test de grossesse

Les femmes enceintes et les femmes en âge de procréer sans contraception en cours sont *a priori* exclues de l'étude. Pour toutes les femmes entrant dans l'étude, un test de grossesse sera réalisé par précaution sur le sérum prélevé à J1. L'inclusion sera effectuée sans attendre le résultat du dosage de la créatininémie. Une valeur >120 entraînera l'arrêt du traitement par la chloroquine et l'exclusion du patient de la suite de l'étude.

14.2.3 Informations recueillies par autoquestionnaire (patient)

Autosuivi à J1 puis deux fois par jour jusqu'à J5, une fois par jour jusqu'à J14 :
Température, arthralgies, asthénie, céphalées, myalgies, prises médicamenteuses, observance (jusqu'à J5), recueil chaque jour de la capacité à faire les activités normales, état général, qualité du sommeil (sur échelle de 10 cm visuelle)(voir Annexe).

14.2.4 Informations recueillies par l'équipe mobile de monitorage

Il s'agit des recueils effectués sur site à J1, J3, J6 et J16 par l'équipe mobile de monitorage.

- Prélèvement sanguin à J1 (avant l'administration du médicament de l'essai), J3, J6, J16
- Fiche à remplir par le clinicien préleveur à chaque visite :
  - Date, heure et lieu du prélèvement
  - Données démographiques du patient (numéro d'identification, DNN, sexe, profession, lieu de résidence)
  - Identification du type de prélèvement: prélèvement à l'inclusion ou en post inclusion ;
  - Identité du préleveur
  - Type de prélèvement

  - Difficultés liées à la réalisation du prélèvement
  - Notion d'épidémie familiale à l'inclusion

Sous forme d'une fiche à remplir par le TEC préleveur à chaque visite :

- Date, heure et lieu du prélèvement
- Données démographiques du patient (numéro d'identification , DNN, sexe, profession, lieu de résidence)
- Identification du type de prélèvement: prélèvement à l'inclusion ou en post inclusion ; identification de l'inclusion du patient dans la sous-étude virologique ; RT-PCR quantitative ; chloroquinémie ; sérologie (IgG, IgM) ; dosage de cytokines (IL1Ra, IL6, IL12p40, RANTES, MCP-1)
- Identité du préleveur
- Difficultés liées à la réalisation du prélèvement
